# Copper/MYC/CTR1 interplay: a dangerous relationship in hepatocellular carcinoma

**DOI:** 10.18632/oncotarget.24282

**Published:** 2018-01-20

**Authors:** Cristiana Porcu, Laura Antonucci, Barbara Barbaro, Barbara Illi, Sergio Nasi, Maurizio Martini, Anna Licata, Luca Miele, Antonio Grieco, Clara Balsano

**Affiliations:** ^1^ MESVA Department, University of L’Aquila, L’Aquila, Italy; ^2^ F. Balsano Foundation, Rome, Italy; ^3^ Institute of Molecular Biology and Pathology, National Research Council, Rome, Italy; ^4^ DIBIMIS, University of Palermo, School of Medicine, Palermo, Italy; ^5^ Fondazione Policlinico Universitario Gemelli, Università Cattolica del Sacro Cuore, Rome, Italy

**Keywords:** hepatocellular carcinoma, copper, non alcoholic fatty liver disease, MYC, CTR1

## Abstract

Free serum copper correlates with tumor incidence and progression of human cancers, including hepatocellular carcinoma (HCC). Copper extracellular uptake is provided by the transporter CTR1, whose expression is regulated to avoid excessive intracellular copper entry. Inadequate copper serum concentration is involved in the pathogenesis of Non Alcoholic Fatty Liver Disease (NAFLD), which is becoming a major cause of liver damage progression and HCC incidence. Finally, MYC is over-expressed in most of HCCs and is a critical regulator of cellular growth, tumor invasion and metastasis.

The purpose of our study was to understand if higher serum copper concentrations might be involved in the progression of NAFLD-cirrhosis toward-HCC. We investigated whether high exogenous copper levels sensitize liver cells to transformation and if it exists an interplay between copper-related proteins and MYC oncogene.

NAFLD-cirrhotic patients were characterized by a statistical significant enhancement of serum copper levels, even more evident in HCC patients. We demonstrated that high extracellular copper concentrations increase cell growth, migration, and invasion of liver cancer cells by modulating MYC/CTR1 axis. We highlighted that MYC binds a specific region of the CTR1 promoter, regulating its transcription. Accordingly, CTR1 and MYC proteins expression were progressively up-regulated in liver tissues from NAFLD-cirrhotic to HCC patients.

This work provides novel insights on the molecular mechanisms by which copper may favor the progression from cirrhosis to cancer. The Cu/MYC/CTR1 interplay opens a window to refine HCC diagnosis and design new combined therapies.

## INTRODUCTION

Copper (Cu) homeostasis is emerging as crucial in many metabolic disorders, as well as in cancer [1–4]. Cu can be found in a oxidized (Cu II) and a reduced (Cu I) state: the latter is transported inside the cell by the high affinity copper transporter CTR1, predominantly found on plasma membrane, and encoded by the *SLC31A1* gene [5, 6]. The two different forms of Cu are important for cellular antioxidant defense and mitochondrial respiration [7]. Free copper is mainly bound to the metal-binding protein ceruloplasmin (Cp), primarily synthesized in the liver [8]. Copper, in its “free” and “unbound” form, becomes toxic by acting as pro-oxidant, contributing to the formation of toxic reactive oxygen species (ROS) and altering the functions of some important biomolecules (i.e. lipids and proteins) [9]. Accordingly, Cu metabolism results significantly altered in chronic and neoplastic diseases [10–11]. Interestingly, serum Cu concentration correlates with hepatocellular carcinoma (HCC) incidence and progression [12].

Growing evidences suggest that, in western countries, NAFLD is becoming a major cause of liver damage progression and HCC incidence [13]. Increased oxidative stress is considered a key trigger in the pathogenesis of this disease and one of the enzymes counteracting oxidative stress, Cu/Zn superoxide dismutase (SOD) depends on adequate copper availability, suggesting a potential link between copper and impaired antioxidant defense in NAFLD. The majority of deaths in patients with NAFLD are, first, attributed to cardiovascular events, and, second to malignancies at gastrointestinal site (liver, colon, esophagus, stomach, and pancreas), while end-stage liver disease is the third cause of death [14].

Most of HCC patients are diagnosed at advanced stages-despite the impressive improvements in imaging techniques-and are characterized by a poor prognosis [15, 16]. Thus, new biomarkers with better diagnostic potential, as well as prognostic value for the assessment of the progression of cirrhosis to HCC, are urgently needed. Furthermore, despite the huge number of studies attempting to improve treatments, currently there are not specific anti-tumoral therapies effective for HCC patients, ineligible to radical treatments. Sorafenib, an orally active multikinase inhibitor, is the only approved drug in the European Union for patients with advanced HCC, who are not candidates for potentially curative treatment or transcatheter arterial chemoembolization (TACE), but unfortunately it prolongs survival for less than 3 months [17]. Therefore, new information on HCC pathogenesis will open new opportunities in the diagnosis and design of patient-tailored therapies. Local invasion and metastasis are important manifestations of advanced HCC and are related to the epithelial-mesenchymal transition (EMT), one of the key processes of tumor progression [18]. The main feature of EMT is the decrease of cell adhesion molecules, such as E-cadherin, and the increase of cytoskeletal proteins like β-catenin, giving to cells mesenchymal like morphology [19]. Through EMT, epithelial cells lose polarity and decrease the connection with the basement membrane, acquiring the ability to migrate and invade the surrounding tissue [18].

c-MYC (MYC) has a pivotal role in cell transformation and EMT modulation [20, 21] and represents one of the most relevant targets for cancer treatment, as demonstrated by studies in animal models employing Omomyc, a MYC interfering molecule [22], or employing drugs that affect MYC transcription [23].

Here, we looked at copper levels in NAFLD-cirrhotic and -HCC patients, highlighting higher serum copper concentrations in presence of liver cancer. Furthermore, we studied the biological effects of growing extracellular copper amounts on HepaRG and HepG2 liver cell lines.

Our data highlight a still unknown interplay between copper, MYC and CTR1.

## RESULTS

### Concentration of copper in serum of NAFLD-cirrhotic and -HCC patients

**Figure 1:**
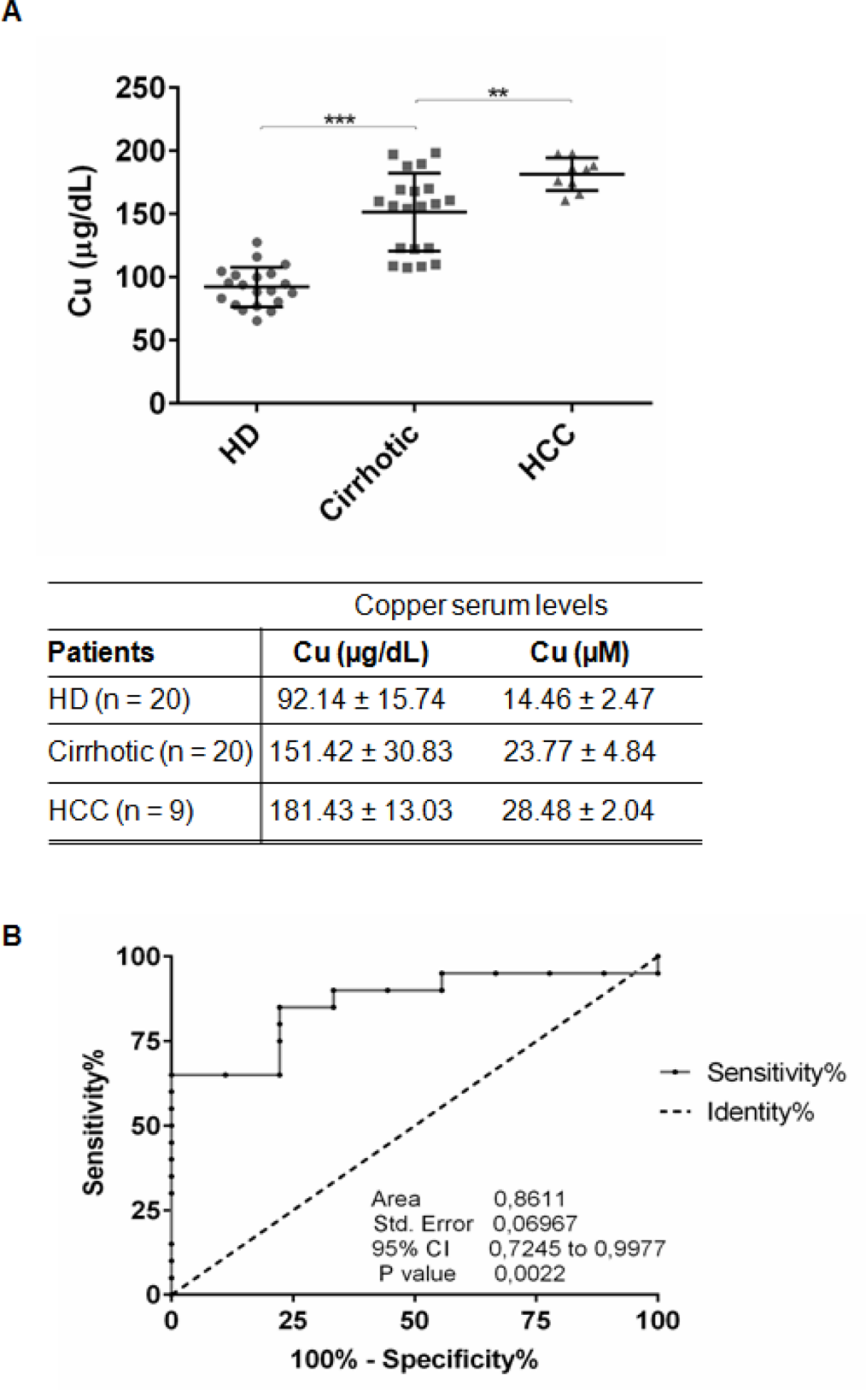
Serum copper level in cirrhotic and HCC patients (**A**) Copper level in serum of HD (*n* = 20), Cirrhotic (*n* = 20) and HCC (*n* = 9) patients quantified by atomic absorption spectroscopy. The results are represented as mean values ± SD. (^**^P < 0.01 and ^***^P < 0.001). (**B**) Sensitivity and Specificity ROC curve based on copper serum levels in HCC versus cirrhotic patients.

We measured copper concentration in sera of 20 NAFLD-cirrhotic, 9 NAFLD-HCC patients and 20 control healthy donors (HD) (Figure 1A, Table 1). NAFLD-cirrhotic patients were characterized by a statistical significant enhancement of serum copper levels compared to HD (151.42 ± 30.83 μg/dl vs 92.14 ± 15.74 μg/dl; *P* < 0.001), and a further significant increase of cupremia was detected in presence of HCC (181.43 ± 13.03 μg/dl vs cirrhotic patients; *P* < 0.01) (Figure 1A). ROC curve analysis revealed that copper could be considered a biomarker to distinguish HCC from cirrhotic patients, since the area under ROC curve (AUC) was 0.8611 (95% CI, 0.7245–0.9977; *P* < 0.01). We identified 163.3 μg/dl as copper cut off value with 65% of Sensitivity and 88.89% of Specificity (Figure 1B).

**Table 1:** Antropometric and serum biochemical parameters

Parameters	HD (*n* = 20)	Cirrhotic (*n* = 20)	HCC (*n* = 9)
Sex (F/M)	7/13	8/12	1/8
Age (years)	41.65 ± 10.95	64.8 ± 9.7	68.6 ± 8.06
BMI (kg/m2) (≤25)	22.4 ± 1.68	31.7 ± 3.07^***^	25.06 ± 6.2^*^
ALT (10–40 IU/mL)	26.7 ± 6.35	68.73 ± 24.6^***^	40.1 ± 19.9^**^
AST (9–45 IU/mL)	20.86 ± 5.43	32.33 ± 8.9^***^	51.33 ± 42.9^**^
GGT (5–38 IU/mL)	24.5 ± 7.9	70.65 ± 15.27^***^	103.6 ± 80.40^***^
FA (40–129 IU/L)	72.85 ± 37.01	113.6 ± 67.3^*^	290.8 ± 309.5^**^
Cu (μg/dL)	92.14 ± 15.7	151.4 ± 26.8^***^	181.4 ± 13.8^***^

Antropometric and serum biochemical parameters of healthy donors (HD), NAFLD cirrhotic and HCC patients. Abbreviation: F, females; M, males; BMI, Body Mass Index; IU, international units; ALT, alanine transaminase; AST, aspartate transaminase; GGT, Gamma-glutamyl transferase; FA, Alkalinfosfatase; Cu, copper. Brackets contain normal values. *P* value has been calculated respect to HD (^*^P < 0.05; ^**^P < 0.01 and ^***^P < 0.001).

Then, we performed statistical correlations between copper and biochemical parameters of NAFLD-cirrhotic and -HCC patients. Even if a positive trend was highlighted with AST, GGT, FA and a negative trend with ALT and TGs, a significant negative correlation has been appreciated only between copper and cholesterol serum levels (Supplementary Figure 1).

Copper promotes cell proliferation

**Figure 2:**
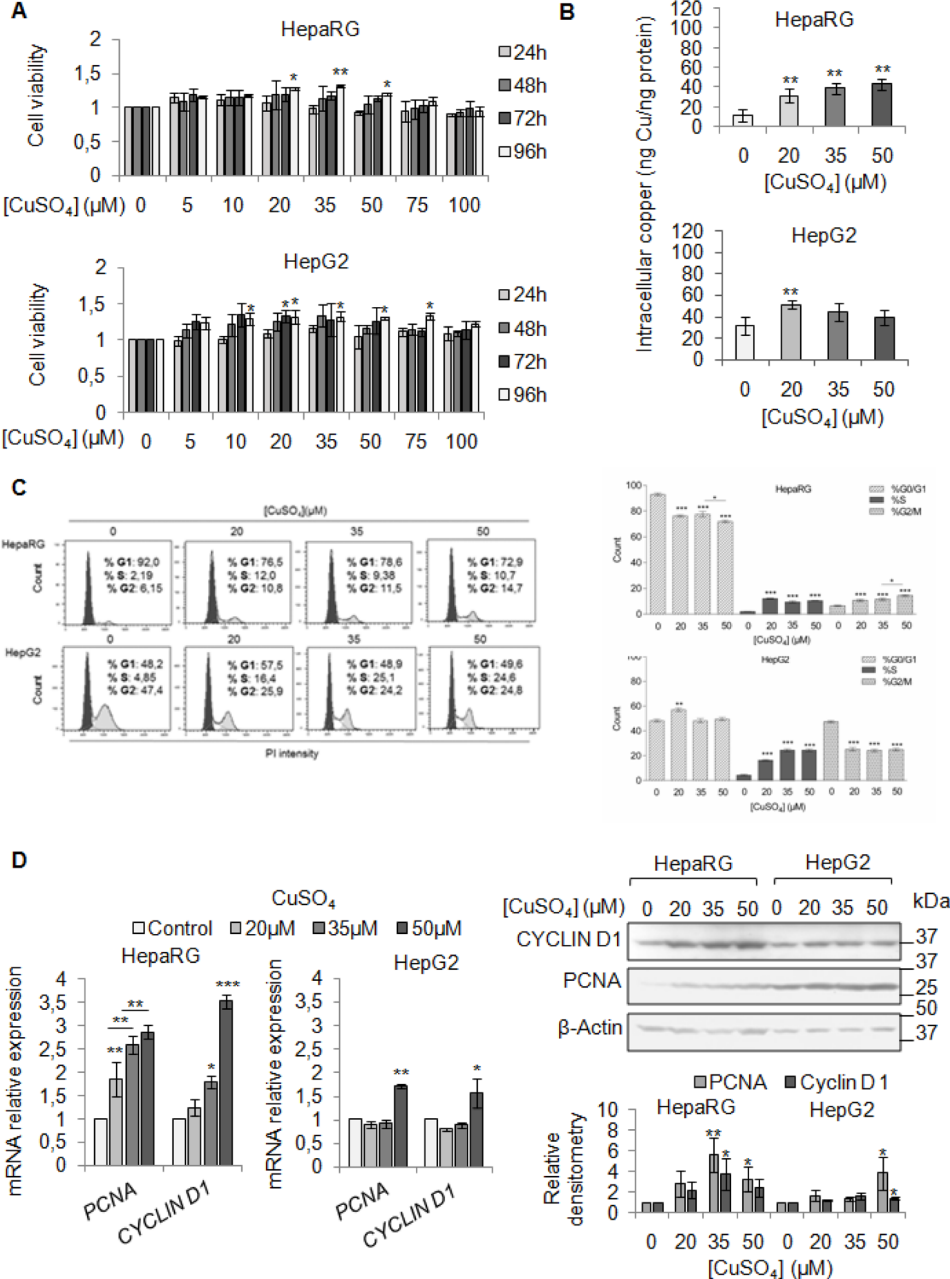
Copper promotes cell proliferation in liver cells (**A**) Relative cell viability evaluated by MTS assay. Relative cell viability of treated cells has been evaluated respect to untreated cells, considered as 0. The results, derived from five independent experiments, are represented as mean ± SD. (**B**) Intracellular copper levels assayed by atomic absorption spectroscopy after 96 hrs of 20, 35 and 50 μM CuSO_4_ treatment. Values are expressed as mean ± SD. (^*^P < 0.05 and ^**^P < 0.01; *n* = 5). (**C**) Left: Representative distribution of control and treated cells with CuSO_4_ (in serum free medium) in G0/G1, S and G2/M phases of cell cycle done by Propidium Iodide (PI) staining and flow cytometric analysis. Right: Histograms reported the values as mean ± SD. (^*^P < 0.05; ^**^P < 0.01; ^***^P < 0.001; *n* = 3). (**D**) Relative mRNA expression (Left), representative western blot (Top, Right) and the relative densitomentric analysis (Bottom, Right) of PCNA and Cyclin D1 in Control and copper treated cells. Values are expressed as mean ± SD. (^*^P < 0.05 and ^**^P < 0.01; *n* = 3).

We firstly evaluated the effect of exogenous copper on viability of HepaRG and HepG2 cells. We performed a dose/time curve with different concentrations of CuSO4 (5–100 μM) (Figure 2A). Copper treatment induced an increase of cell viability in both cell lines, especially at 96 hrs (Figure 2A). Based on these data, from now on, we decided to perform all the experiments at 96 hrs.

Since our patients displayed serum copper levels between 20 and 30 μM, and the best CuSO_4_ concentrations, in *in vitro* experiments, appeared to be between 20 and 35 μM, we decided to use the following concentrations of CuSO_4_: 20, 35 and 50 μM. Copper treatment induced a significant increase of intracellular copper levels, in both cell lines (Figure 2B). The cell cycle analyses of starved liver cells stimulated by increasing copper concentrations, revealed a higher number of cells in S phase (Figure 2C), without increasing cell death (Supplementary Figure 2). The up-regulation of mRNA and protein levels of PCNA and Cyclin D1 confirmed the pro-proliferative effects of copper (Figure 2D), whereas, the increased number of cells in G2/M was associated with increased expression of Cyclin B1 and Cyclin A (Supplementary Figure 3).

### Copper and MYC interaction

**Figure 3:**
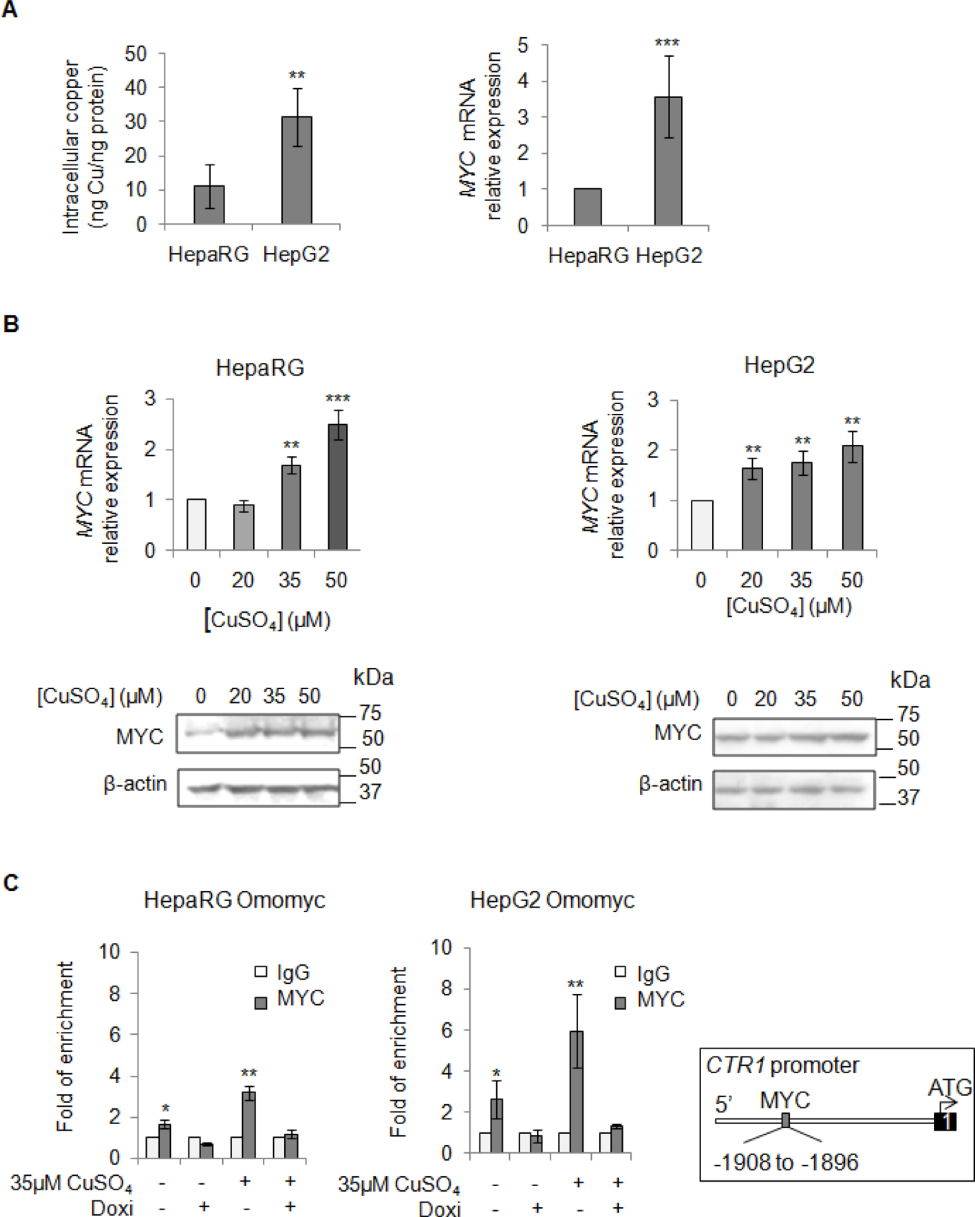
Direct interaction between MYC and CTR1 (**A**) Left: Basal intracellular copper levels assayed by atomic absorption spectroscopy in HepaRg and HepG2 cells. Right: relative mRNA expression of *MYC* in HepG2 and HepaRG cells. Values are expressed as mean ± SD. (^**^P < 0.01 and ^***^P < 0.001; *n* = 5). (**B**) Relative mRNA expression and representative western blots of MYC in HepaRG (Left) and HepG2 (Right) cells after 20, 35 and 50 μM CuSO_4_ treatment (96 hrs). Values are expressed as fold mean ± SD. (^**^P < 0.01; *n* = 3). (**C**) MYC binding to the *CTR1* promoter region, assayed by ChIP, after induction of Omomyc by doxycyclin (Doxi), before and after treatment with 35 μM CuSO_4_ (96 hrs). Values are expressed as fold mean ± SD of three independent experiments (^*^P < 0.05 and ^**^P < 0.01). IgG was used as negative ChIP control. Right: Schematic representation of the MYC binding consensus sequences on the *CTR1* promoter.

Aware that most of HCCs over-express MYC [24, 25], a critical regulator of cellular growth, we looked at MYC mRNA expression levels before and after copper stimuli (Figure 3A and 3B). We highlighted a positive correlation between basal intracellular levels of copper and MYC expression (Figure 3A), as well as an induction of MYC expression after copper stimuli (Figure 3B). Densitometric analyses for all the immunoblots presented in this manuscript has been reported in supplementary data (Supplementary Figure 4).

However, since copper is important for cellular antioxidant defense and mitochondrial respiration, to further improve the relevance of specific copper-related effects on MYC induction, we evaluated, in both cell lines, the extent of oxidative stress in presence of increasing copper concentrations (20–100 μM CuSO4) (Supplementary Figure 5). Copper treatment induced an increase of MYC protein amount (Supplementary Figure 5A) that was associated with an increase of ROS (Supplementary Figure 5B and 5C), detected by using 2′, 7′-dichlorodihydrofluorescein diacetate (H2DCF-DA) fluorescent probe.

After that, we investigated the effect of Hydrogen peroxide (H_2_O_2_) on MYC expression in correlation with ROS production (Supplementary Figure 5E and 5F). Interestingly, H2O2 treatment, contrary to copper, determined a relevant down-regulation of MYC protein levels in HepaRG cells (Supplementary Figure 5D).

These data prompted us to study if MYC could modulate the expression of CTR1, the main copper transporter protein responsible for the influx of reduced copper ions across cell membrane. Thus, we investigated if MYC might bind to the CTR1 promoter region (Figure 3C). For this purpose, liver cell lines were stably transfected with Omomyc, a dominant negative of MYC, here used as negative control. Omomyc was chosen because it impairs the bind of MYC/MAX complex to DNA-recognizing E-boxes -, thus working as a transcriptional inhibitor [22]. After that, we performed a Chromatin Immunoprecipitation (ChIP) assay, using a specific anti-MYC antibody to precipitate chromatin fragments from controls and Omomyc HepaRG and HepG2 cells, in presence or absence of exogenous copper. Copper caused a higher MYC binding to CTR1 promoter (3.2 and 5.9 folds in HepaRG and HepG2 cells, respectively) respect to the control, while any binding was observed in presence of Omomyc (Figure 3C). These results highlighted a direct interaction between MYC and *CTR1* promoter, mediated by the MYC binding consensus sequence (AGAGCACATGGCT) located between −1908 and −1896 of the transcription start site of *CTR1* promoter region.

To understand how spread is the copper activity on MYC, we tested beyond CTR1, by ChIP experiments, other c-MYC target genes, known to be important for cell proliferation and invasion: CAD (carbamoyl-phosphate synthetase 2) and Cyclin D1 proteins. Copper treatment increased MYC binding at CAD and Cyclin D1 promoters respect to the control, while this interaction was absent in presence of Omomyc (Supplementary Figure 6).

### CTR1 and MYC expression in *in vitro* and *in vivo* experiments

**Figure 4:**
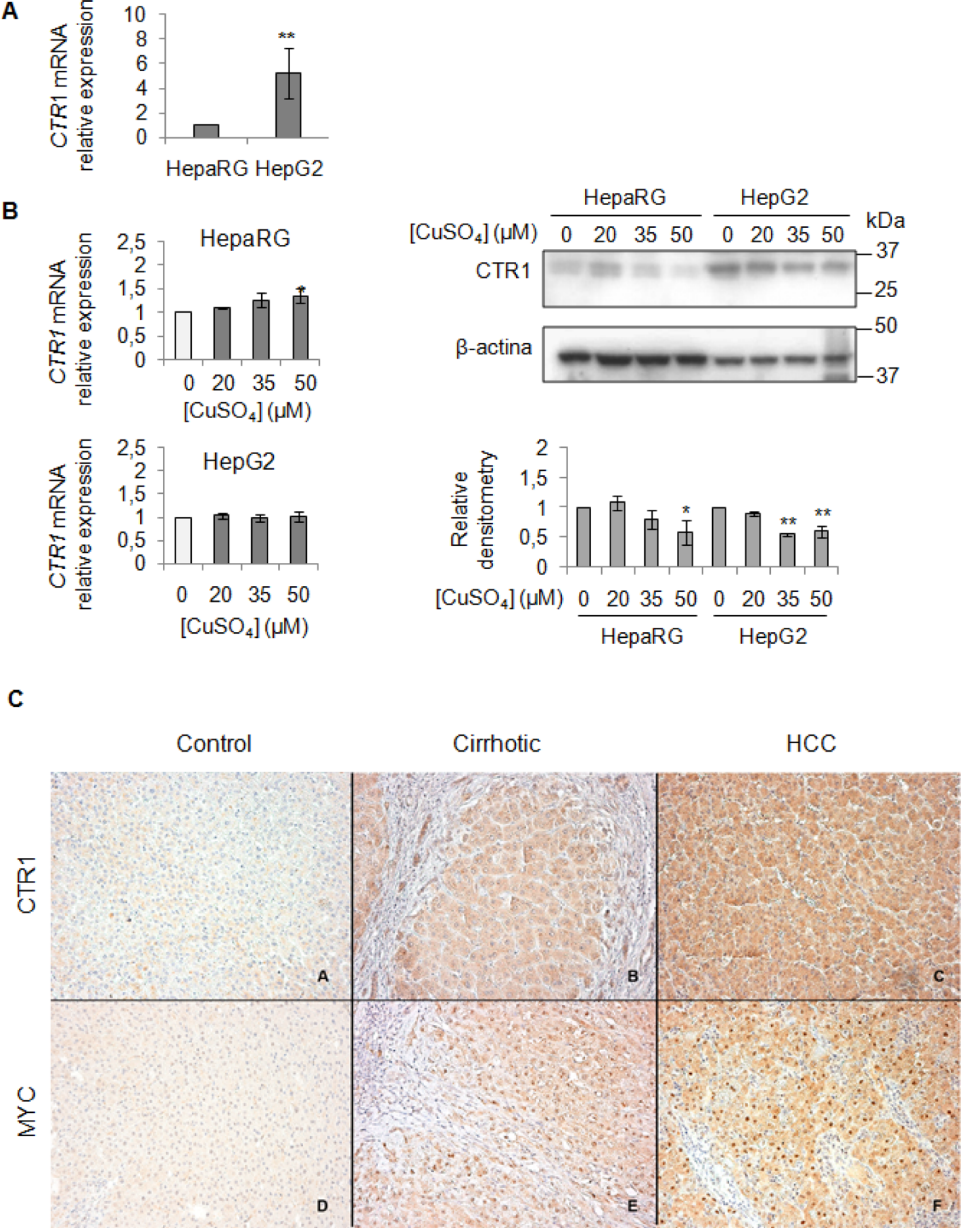
Exogenous copper modulates CTR1 and MYC protein expression (**A**) Relative mRNA expression of *CTR1* measured by RT-PCR in HepG2 respect to HepaRG. Values are expressed as fold mean ± SD. (^**^P < 0.01; *n* = 5). (**B**) Left: Relative mRNA expression of *CTR1* in both cells after 96 hrs of copper treatment. Right: Representative western blot of CTR1 and relative densitometry of three independent experiments. Values are expressed as fold mean ± SD. (^*^P < 0.05 and ^**^P < 0.01; *n* = 3). (**C**) Immunohistochemical analysis of CTR1 and MYC protein expression. Panels A and D: two representative cases of normal liver tissue; Panels B and E: liver cirrhotic tissues. Panel C and F: hepatocellular carcinoma tissues. Avidin-Biotin-Peroxidase complex method in paraffin sections lightly counterstained with ematoxylin. Original magnification 200×

We evaluated the baseline gene expression of *CTR1* in the two cell lines, which resulted higher in HepG2 than in HepaRG (Figure 4A). Unexpectedly, copper treatment caused an increase of *CTR1* transcription only in HepaRG, but not in HepG2 cells (Figure 4B). However, since it is well known that CTR1 protein is down-regulated to avoid excessive intracellular copper entry, accordingly with data reported in literature [26], copper, at the highest concentrations (35 and 50 μM), determined a down-regulation of the CTR1 protein levels, in both cell lines (Figure 4B). Further studies are needed to understand the fine differences existing between less or more differentiated transformed liver cells, such as HepG2 and HepaRG cells.

To validate the role of the MYC/CTR1 axis in HCC, we assessed the expression of MYC and CTR1 in normal, NAFLD-cirrhotic and -HCC tissues by using immunohistochemical staining (Figure 4C). Normal liver tissue expressed very low level of CTR1 (Figure 4C, panel A), whereas MYC was undetectable (Figure 4C, panel D). CTR1 expression progressively increased either in cell membrane or in the cytoplasm from cirrhotic (Figure 4C, panel B) toward HCC tissues (Figure 4C, panel C). On the other hand, MYC protein displayed moderate nuclear expression in cirrhotic tissues (Figure 4C, panel E) that strongly increased in HCC tissues (Figure 4C, panel F).

**Figure 5:**
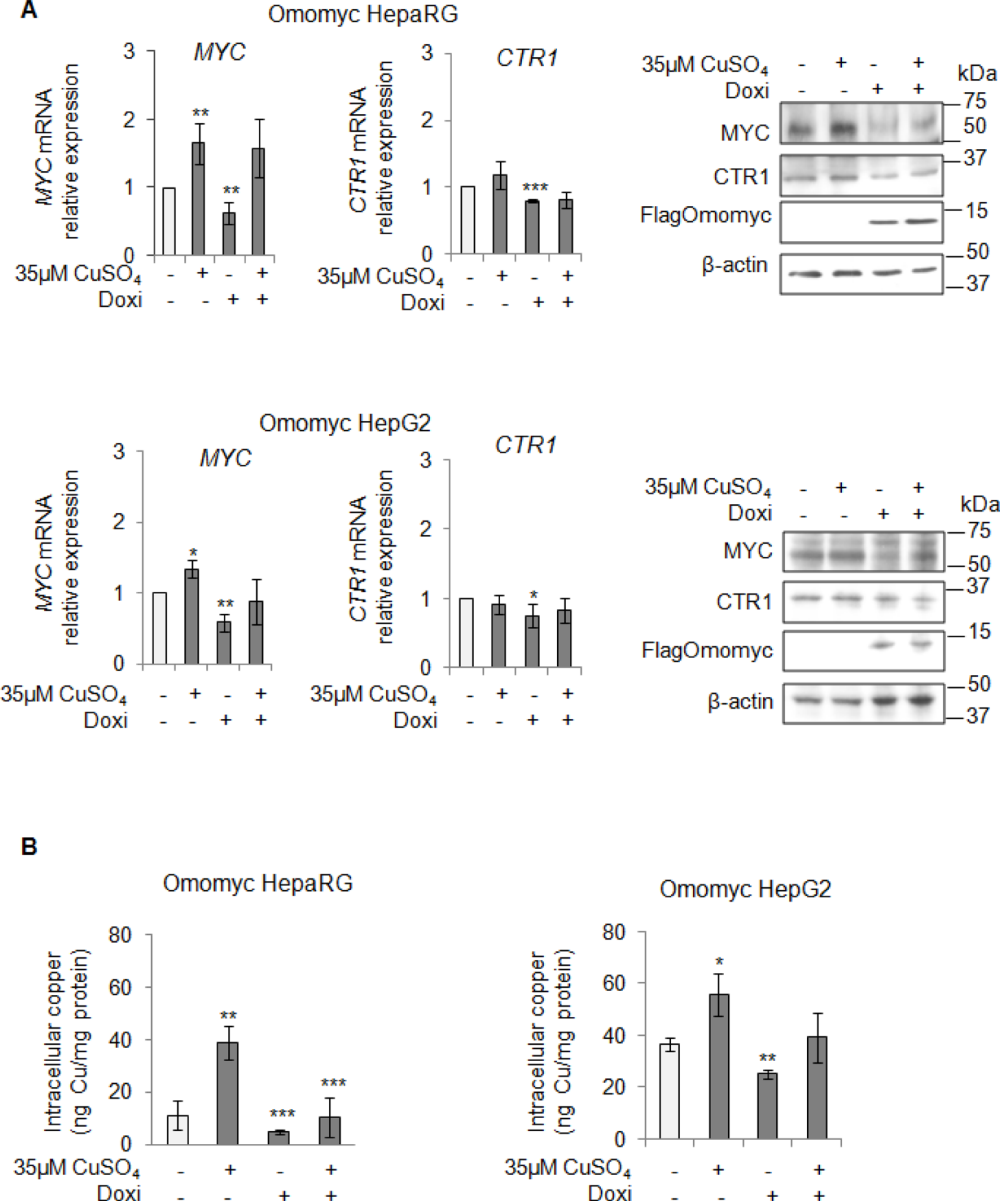
MYC modulates the intracellular copper homeostasis (**A**) Relative mRNA expression (Left) and representative western blots (Right) of MYC, CTR1 and FlagOmomyc in Omomyc HepaRG (Top) and HepG2 (Bottom) cells before and after induction of Omomyc by doxycyclin (Doxi), treated or not with 35 μM CuSO4 (96 hrs). Values are expressed as fold mean ± SD. (^*^P < 0.05; ^**^P < 0.01 and ^***^P < 0.001; *n* = 3). (**B**) Intracellular copper levels in Omomyc HepaRG (Left) and HepG2 (Right) cells in the same experimental condition described above. Values are expressed as fold mean ± SD. (^*^P < 0.05; ^**^P < 0.01 and ^***^P < 0.001; *n* = 3).

After that, we looked at CTR1 expression in stable Omomyc expressing cells. Notably, the CTR1 mRNA and protein amounts decreased in parallel with the reduction of MYC expression (Figure 5A), causing in turn a significant decrease of intracellular copper concentrations (Figure 5B). However, if copper was added again, despite the presence of Omomyc, it was able to partially restore the expression of MYC and to slightly increase the intracellular copper content (Figure 5A and 5B, respectively). Interestingly, stable Omomyc expressing cells displayed a significant decrease of the intracellular copper concentrations (Figure 5B).

### Omomyc partially counteracts the copper related biological effects

**Figure 6:**
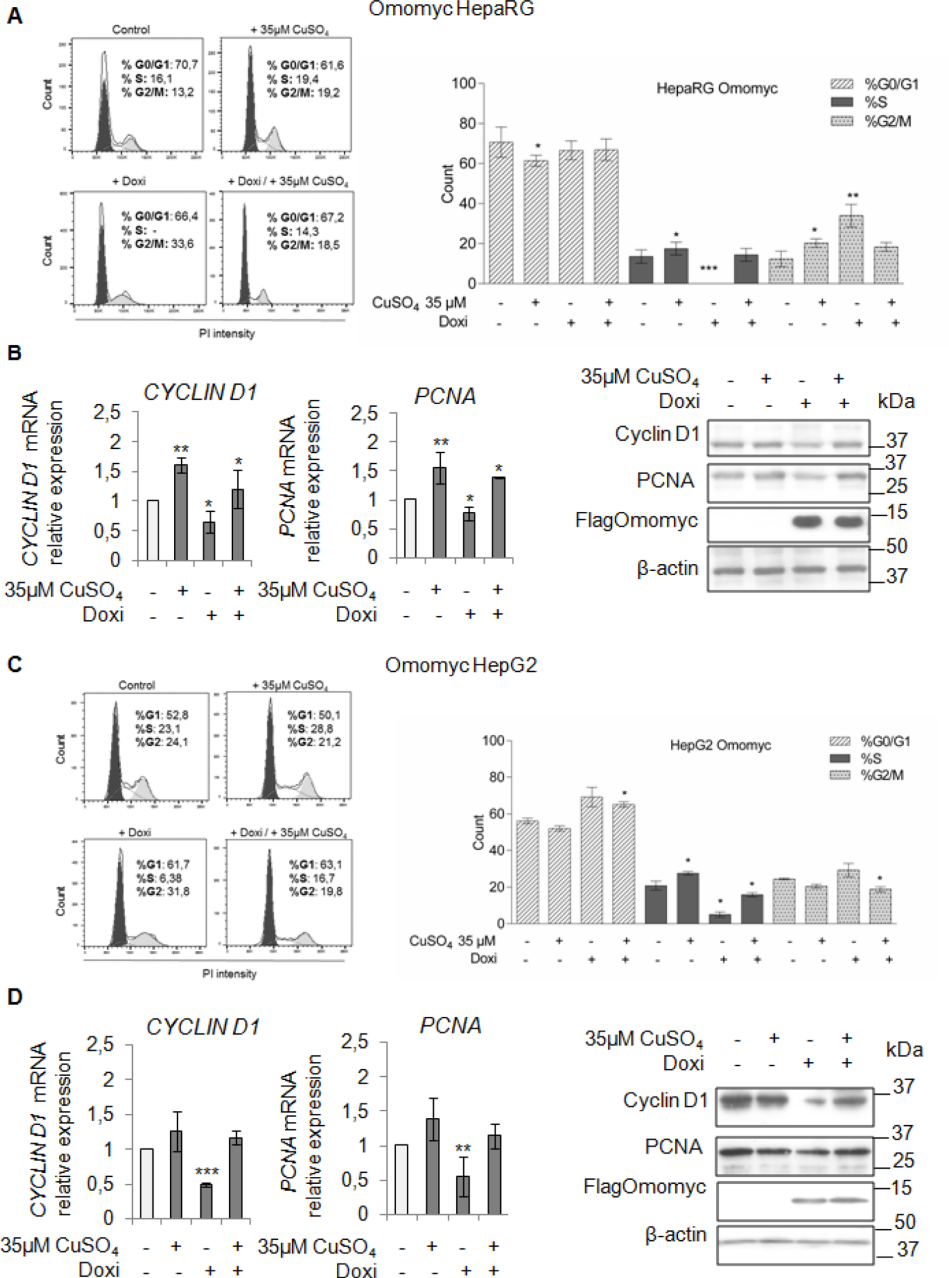
Omomyc and copper-induced proliferation (**A** and **C**) Left: Representative distribution plot of HepaRG (A) and HepG2 (C), after induction of Omomyc by doxycyclin (Doxi), before and after treatment with 35 μM CuSO_4_ (96 hrs), in G0/G1, S and G2/M phases of the cell cycle analysed by PI staining and flow cytometric analysis. Right: Histograms reported values as mean ± SD (^*^P < 0.05; ^**^P < 0.01; ^***^P < 0.001; *n* = 3). (**B** and **D**) Relative mRNA expression (Left) and representative western blots (Right) of Cyclin D1, PCNA and FlagOmomyc in HepaRG (B) and HepG2 (D) cells in the same experimental condition described above (^*^P < 0.05; ^**^P < 0.01; ^***^P < 0.001; *n* = 3).

To understand if the inhibition of the copper/MYC interplay might be effective in lessening the copper-related tumorigenic effects, firstly, we looked at the ability of Omomyc in counteracting the copper-related increased proliferation of HepaRG and HepG2 cells. In both cell lines, Omomyc caused an accumulation of cells in G2/M phase and a decrease of cells in S phase (Figure 6A and 6C). A further stimulus with 35 μM CuSO_4_, partially restored proliferation in both cell lines, regardless the presence of Omomyc (Figure 6A and 6C). Accordingly, PCNA and Cyclin D1 mRNA and protein levels (Figure 6B and 6D) were down-regulated by Omomyc, but upon copper treatment their expression was up-regulated again.

**Figure 7:**
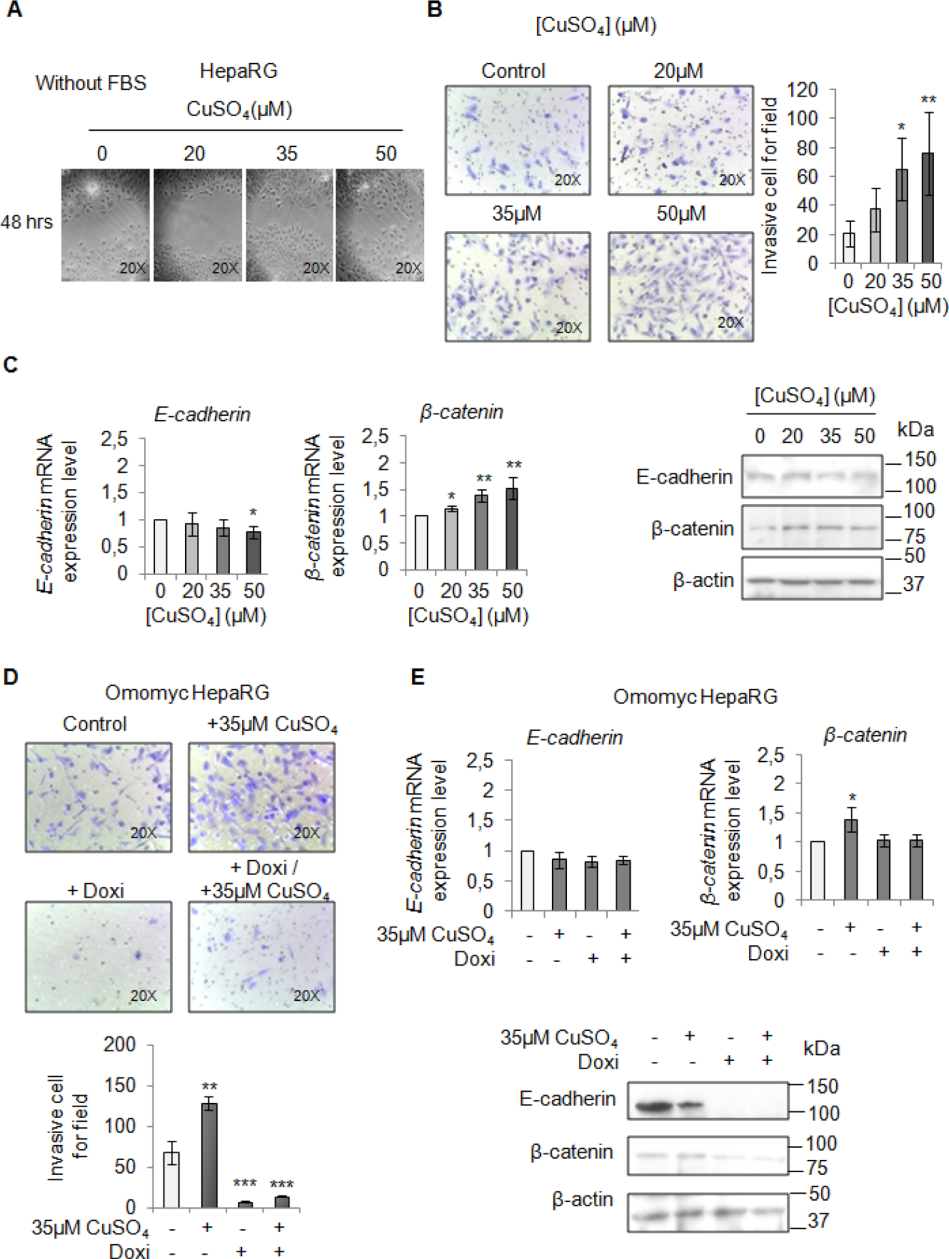
Copper promotes HepaRG invasion by MYC (**A**) Analysis of cell migration by a wound-healing assay in control and CuSO_4_ treated HepaRG cells. Representative microphotographs taken at 48 hrs post-wound (×20). (**B**) Transwell migration assay in HepaRG cells treated with copper for 48 hrs, in serum free medium. Left: Representative microphotographs of crystal violet stained cells migrated to the bottom membrane of transwell (×20). Right: Quantification of the number of migratory cells, that were counted in 5 non-overlapping random fields of the membrane. Values are expressed as fold mean ± SD. (^*^P < 0.05 and ^**^P < 0.01; *n* = 3). (**C**) Relative mRNA expression (Left) and representative western blots (Right) of E-cadherin and β-catenin in HepaRG treated with CuSO_4_ up to 96 hrs. Values are expressed as fold mean ± SD. (^*^P < 0.05; *n* = 3). (**D**) Transwell migration assay in Omomyc HepaRG cells performed in a serum-supplemented medium, after induction of Omomyc by doxycyclin (Doxi), before and after treatment with 35 μM CuSO_4_ (48 hrs). Top: Representative microphotographs of crystal violet stained cells attached to the bottom membrane of a transwell (×20). Bottom: Quantification of the number of migratory cells using the transwell assay. Migratory cells were counted in five non-overlapping random fields of the membrane. Values are expressed as fold mean ± SD. (^*^P < 0.05 and ^***^P < 0.001; *n* = 3). (**E**) Relative mRNA expression (Top) and representative western blots (Bottom) of E-cadherin and β-catenin in Omomyc HepaRG cells treated or not with copper treatment (96 hrs). Values are expressed as fold mean ± SD (^*^P < 0.05; *n* = 3).

**Figure 8:**
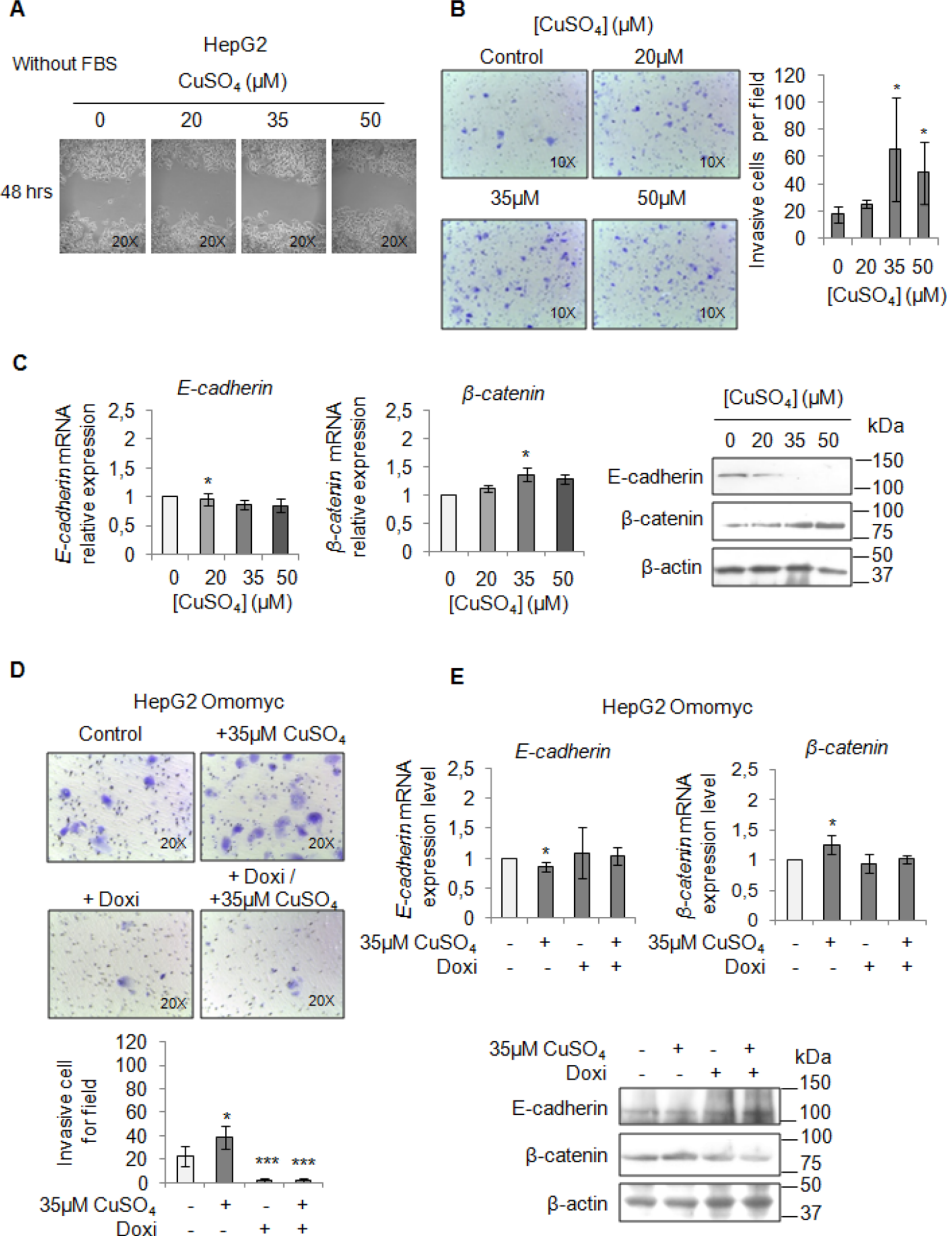
Copper induces HepG2 migration by MYC (**A**) Analysis of cell migration by a wound-healing assay in Control and CuSO_4_ treated HepG2 cells. Representative microphotographs taken at 48 hrs post-wound (×20). (**B**) Transwell migration assay in HepG2 cells treated with copper for 48 hrs in serum free medium. Left: Representative microphotographs of crystal violet stained cells attached to the bottom membrane of a transwell (×20). Right: Quantification of the number of migratory cells, that were counted in five non-overlapping random fields of the membrane. Values are expressed as fold mean ± SD. (^*^P < 0.05; *n* = 3). (**C**) Relative mRNA expression (Left) and representative western blots (Right) of E-cadherin and β-catenin in HepG2 treated with CuSO_4_ for 96 hrs. Values are expressed as fold mean ± SD. (^*^P < 0.05; *n* = 3). (**D**) Transwell migration assay in HepG2 Omomyc cells in a serum-supplemented medium, after induction of Omomyc by doxycyclin (Doxi), before and after treatment with 35 μM CuSO_4_ (48 hrs). Top: Representative microphotographs of crystal violet stained cells attached to the bottom membrane of a transwell (×20). Bottom: Quantification of the number of migratory cells, that were counted in five non-overlapping random fields of the membrane. Values are expressed as fold mean ± SD. (^*^P < 0.05 and ^***^P < 0.001; *n* = 3). (**E**) Relative mRNA expression (Top) and representative western blots (Bottom) of E-cadherin and β-catenin in HepG2 Omomyc inducing cells in presence or not of copper treatment for 96 hrs. Values are expressed as fold mean ± SD (^*^P < 0.05; *n* = 3).

Finally, we performed a migration and invasion assay in starved cells to observe if copper by itself could promote cell mobilization, too. Copper treatment pushed HepaRG (Figure 7A and 7B) and HepG2 cells (Figure 8A and 8B) to migrate and to invade the extracellular matrix. Accordingly, E-cadherin was decreased while β-catenin was up-regulated (Figure 7C and Figure 8C). In addition, the transcription of c-KIT, THY1 and NCAM, specific proteins related to the Epithelial Mesenchimal Transition (EMT) in HepaRG cells, was up-regulated (Supplementary Figure 7). Omomyc was able, to totally revert the copper-dependent pro-invasive effect, in both cell lines, inducing a post-transcriptional regulation of E-cadherin and β-catenin (Figure 7D and 7E, Figure 8D and 8E).

### CTR1 silencing counteracts copper related pro-tumorigenic effects

**Figure 9:**
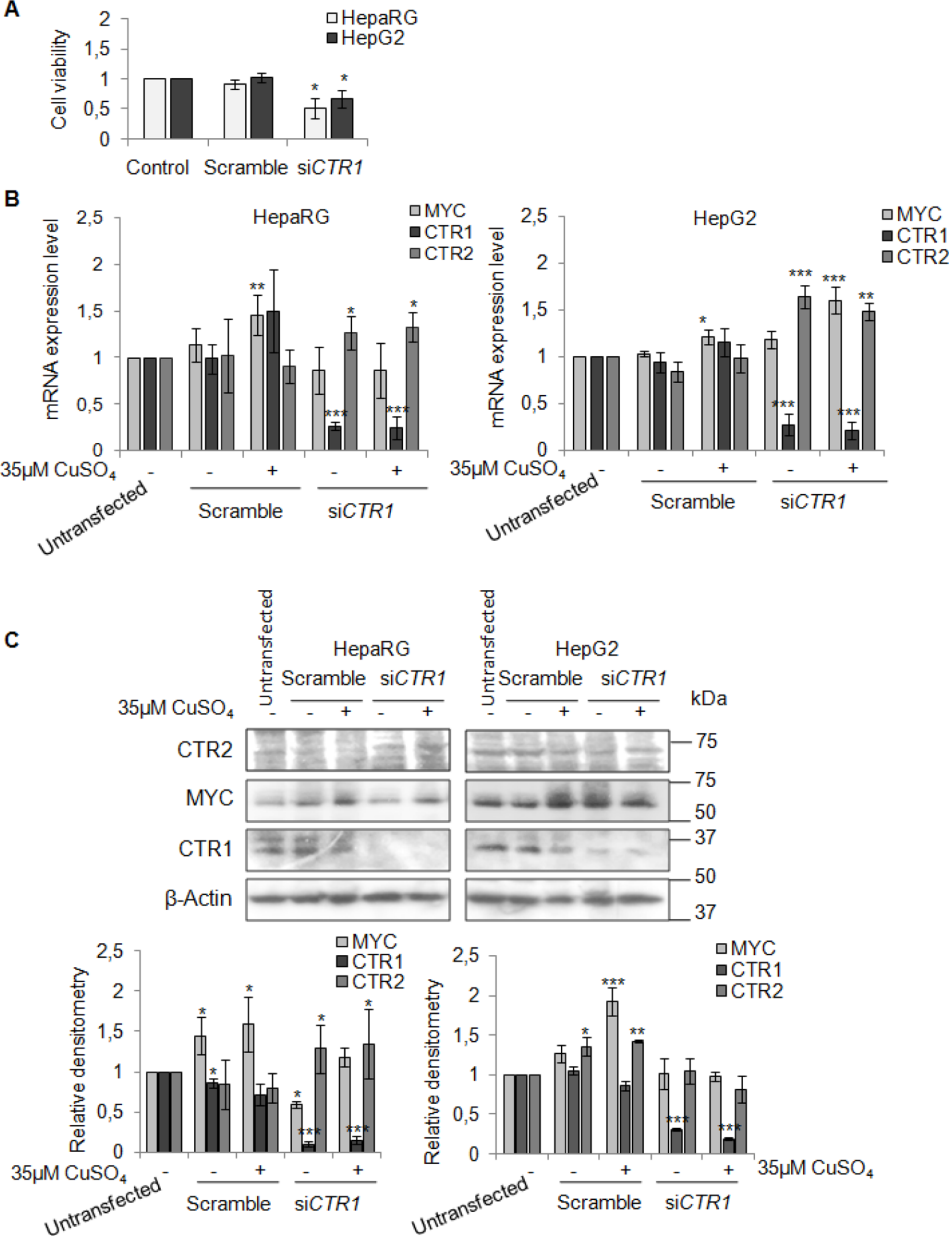
CTR1 silencing (**A**) Relative cell viability evaluated by MTS assay. Results derived from three independent experiments and are represented as mean ± SD, respect to un-transfected cells. (**B**) Relative mRNA expression of *MYC, CTR1* and *CTR2* in scramble and siCTR1 transfected cells, in presence or absence of copper treatment (35 μM CuSO_4_). Values are expressed as mean ± SD (^*^P < 0.05, ^**^P < 0.01, and ^***^P < 0.001, respect to un-transfected cells; *n* = 3). (**C**) Representative western blot (Top) and the relative densitomentric analysis (Bottom) of MYC, CTR1 and CTR2. Values are expressed as mean ± SD (^*^P < 0.05, ^**^P < 0.01, and ^***^P < 0.001, respect to un-transfected cells; *n* = 3).

To better understand the involvement of CTR1 in copper/MYC axis we transfected HepaRG and HepG2 cells with a pool of CTR1 siRNAs (siCTR1). As shown in Figure 9 siCTR1 specifically inhibited CTR1 expression, in presence or absence of 35 μM CuSO_4_. Copper transporter 2 protein, CTR2, was used as control.

**Figure 10:**
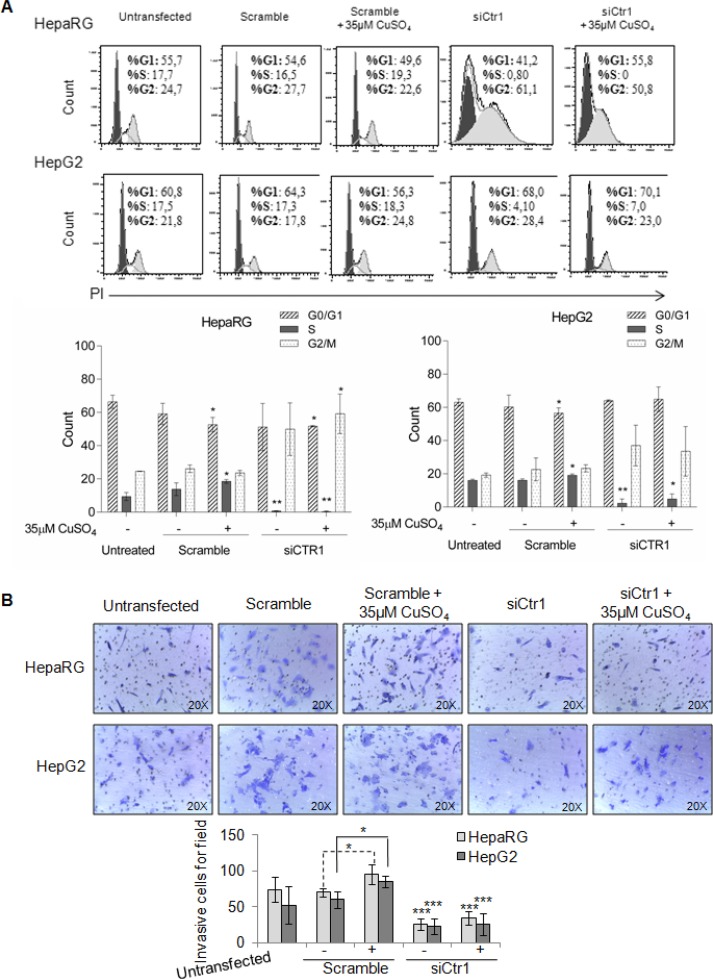
Biological effects of siCTR1 (**A**) Top: Representative cell cycle distribution of un-trasfected, transfected, scramble and siCTR1 cells, in presence or not of 35 μM CuSO_4_ treatment. Cell cycle was done by Propidium Iodide (PI) staining and flow cytometry analysis. Bottom: Histograms reported the values as mean ± SD (^*^P < 0.05; ^**^P < 0.01, respect to untreated cells; *n = 3*). (**B**) Transwell migration assay of HepaRG and HepG2 cells performed after 48 hrs in un-trasfected, transfected, scramble and siCTR1 cells, with or without CuSO_4_ treatment. Top: Representative microphotographs of crystal violet stained cells migrated to the bottom membrane of transwell (×20). Bottom: Quantification of the number of migratory cells, counted in 5 non-overlapping random fields of the membrane. Values are expressed as fold mean ± SD. (^*^P < 0.05 and ^**^P < 0.01; *n* = 3).

In HepaRG cells, CTR1 inhibition caused MYC protein down-regulation, without affecting its transcription. In these cells, MYC expression was restored by copper. On the other hand, siCTR1 didn’t seem to affect MYC protein expression in HepG2, in presence or absence of copper (Figure 9B and 9C). Flow cytometry analysis demonstrated that siCTR1, induced a significant decrease of percentage of cells in S phase and an increase in G2/M phase, in both cell lines. This phenomenon was more evident in HepaRG cells (Figure 10A).

On the other hand, siCTR1 strongly counteracted copper-dependent invasiveness, in both cell lines (Figure 10B).

## DISCUSSION

Beside genetic diseases (e.g. Wilson disease), high levels of copper serum concentration have been shown to correlate with tumor incidence, malignant progression, and recurrence of human cancers, including HCC. Our data indicate that increased circulating Cu significantly correlate with progression of NAFLD-cirrhosis toward -HCC (Figure 1A), suggesting that, as supported by the ROC curve analysis (sensitivity 65% and specificity 88.89%) (Figure 1B), copper might be a new biomarker of hepatocyte transformation. We identified a copper cut off value (163.3 μg/dl) to discriminate NAFLD cirrhotic patients prone to progress toward HCC (Figure 1B). Similar results (data not shown) were obtained in HCV-cirrhotic and -HCC patients, supporting the idea that high serum copper concentration seems to well correlate with the progression of liver cirrhosis to HCC, regardless its etiology. However, to consider copper as a warning for clinicians who follow NAFLD-cirrhotic patients, a longitudinal prospective multicenter clinical trial, devoted to answer if copper could be considered a biomarker of liver cell transformation and/or progression, should be developed.

Our clinical observations pushed us to investigate on the pathogenic involvement of copper in liver tumorigenesis. Copper treatment of HepaRG and HepG2 cells was able to induce their proliferation (Figure 2), migration and invasion (Figure 7 and Figure 8). These effects were associated with the up-regulation of PCNA and Cyclin D1, and with a down-regulation of E-cadherin together with an up-regulation of β-catenin expression levels. These data indicate that high copper concentrations in cell microenvironment have a main role in promoting both the onset and progression of liver tumor.

**Figure 11:**
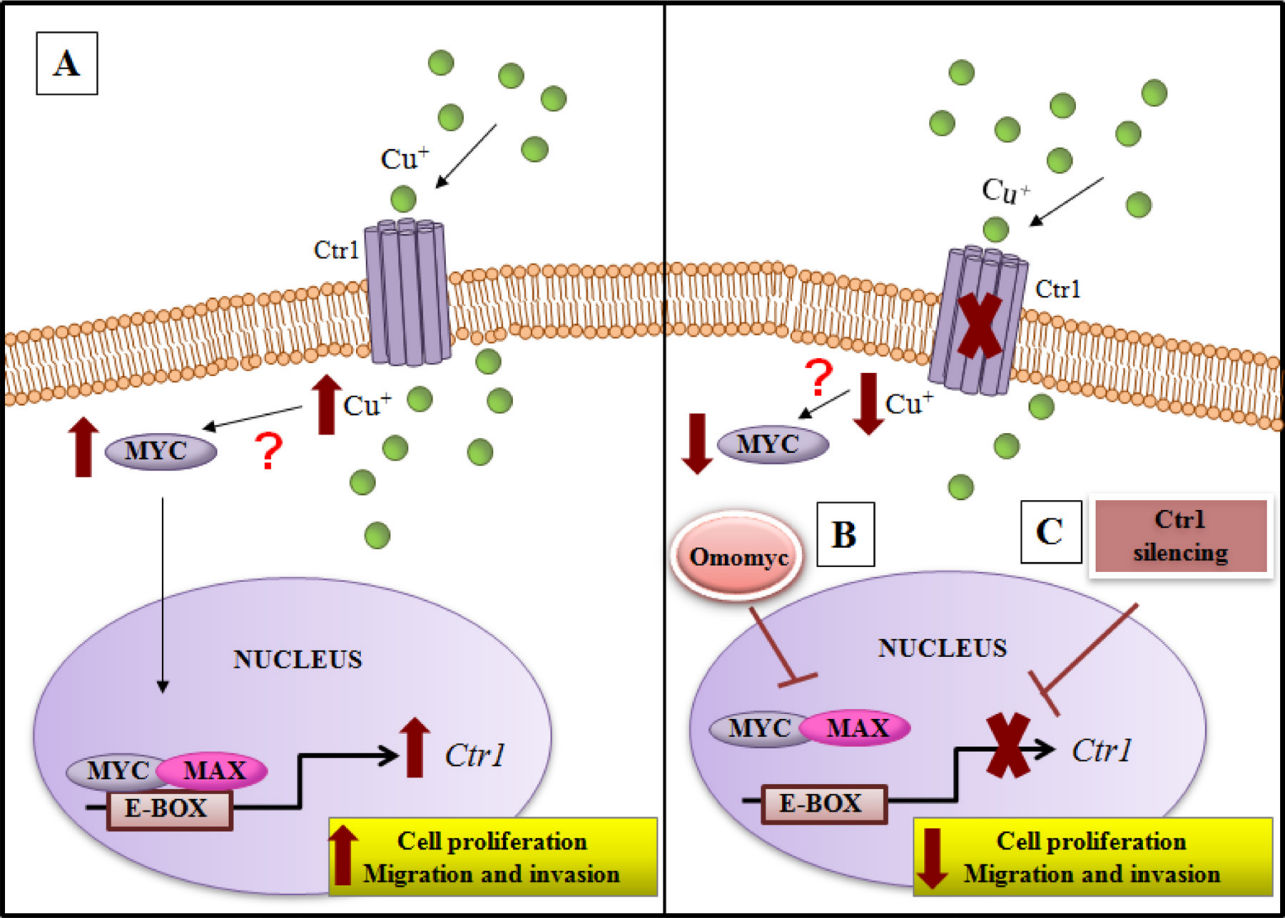
Model of copper/MYC/CTR1 axis in liver cells (**A**) Higher levels of extracellular copper increase MYC expression that in turn by a direct interaction with the CTR1 promoter induces its transcription. The biological effect of MYC/CTR1 interplay promotes cell proliferation and invasiveness by the increased copper intracellular concentration. (**B**) Conversely, Omomyc, a dominant negative of MYC, impairing the bind of MYC/MAX complex to DNA on the CTR1 promoter, is able to counteract the copper-dependent effects, (**C**) as well as CTR1 silencing.

We highlighted, for the first time, that an interplay between copper and MYC exists, underscoring the ability of copper in inducing MYC expression (Figure 3B). Moreover, we demonstrated that MYC binds a specific consensus region on *CTR1* promoter (Figure 3C), the principal copper transporter protein. Exploiting the anti-proliferative and anti-invasive efficacy of Omomyc (Figures 6, 7 and 8), a MYC dominant negative that inhibits the MYC/MAX interaction, we highlighted that MYC/MAX dimer is necessary for the transcription of *CTR1*, influencing, in this way, intracellular copper levels (Figure 5A and 5B). A schematic regulatory model of copper/MYC/CTR1 axis was proposed in Figure 11.

The addition of exogenous copper induced an increase of *CTR1* transcription in HepaRG cells, but not in HepG2 cells (Figure 4B). This fact is maybe due to the high basal levels of copper in HepG2 cells (Figure 4A). Unexpectedly, CTR1 protein was down-regulated in both cell lines after Cu stimulation, suggesting that, when high extracellular Cu raises, cells activate a safeguard mechanism by a rapid internalization and/or degradation of CTR1 [6, 26]. In fact, accordingly with our data (Figure 4A and 4B), it has been recently reported that, in *in vitro* model, CTR1 expression, under high Cu conditions, is down-regulated [27].

Moreover, the CTR1 silencing caused, in HepaRG, a down-regulation of MYC expression, but after copper stimulation was able to restore its expression. No regulation of MYC protein was appreciated in HepG2 cells (Figure 9B and 9C).

Interestingly, our work highlights the differences existing between HepG2 and HepaRG cells. In fact, it seems that HepG2 cells lose the physiological regulation of copper/MYC/CTR1 axis, at least as regards its specific role in regulation of MYC expression. However, these differences are not surprising, in fact, HepaRG cells have been developed to tackle the problem of low metabolic profiles observed in HepG2 cells. Indeed, HepaRG cells retain a drug metabolism capacity comparable to that of primary hepatocytes with a functional stability as well as primary cells [28]. HepaRG, but not HepG2 cells, maintain hepatic functions and expression of liver-specific genes at comparable levels to hepatocytes [29]. Moreover, copper is implicated in handling and utilizing proteins involved in the striking metabolic changes that occur in cancer cells. Copper, in fact, has an essential role in cytochrome oxidase function, thus our results indicate that it would be interesting to ascertain if the expression of proteins that function in copper cellular influx might play a role in the Warburg effect.

Omomyc, a MYC interfering molecule, was partially able to revert the copper-related pro-proliferative effects (Figure 6), while it completely counteracted the copper-dependent invasiveness (Figure 7D and 7E, Figure 8D and 8E). This behavior could be due to the ability of copper in partially restoring the expression of MYC, despite the presence of Omomyc (Figure 5B) or to the participation of other important intracellular pathways that might counterbalance the suppression of MYC.

Finally, the immunohistochemical analysis for CTR1 and MYC proteins performed in normal, NAFLD-cirrhotic and -HCC tissues (Figure 4C), show a progressively increase of both CTR1 and MYC protein expression from normal toward HCC tissues. In line with these observations, the less differentiated HepG2 cells showed, at the baseline, higher CTR1 mRNA and protein levels respect to HepaRG cells (Figure 4A and B).

Our results are consistent with several recent findings on the importance of the microenvironment in defining tumor promotion and aggressiveness [30, 31]. However, the regulatory feedback involved in the control of the copper/MYC/CTR1 axis should be deeply analyzed by further studies.

The Cu/MYC/CTR1 interplay could pave the way to new approaches in refining HCC diagnosis and in planning new combined therapies. In this view, the reduction of the bioavailability of copper through copper chelation therapies could be of support to the standard chemotherapy, counteracting the CTR1/MYC dangerous connection.

## MATERIALS AND METHODS

### Patients and laboratory parameters

The study was performed on 20 control healthy subjects (HD), 20 NAFLD-cirrhotic and 9 NAFLD-HCC patients enrolled in accordance with the ethical guidelines of the 1975 Declaration of Helsinki and approved by Ethical Committee of the Sapienza University, Rome, Italy (N. 2534) and by the other involved Institutions. Informed consent was obtained from all patients.

The diagnosis of NAFLD-cirrhosis and NAFLD-HCC was based on exclusion of other common causes of cirrhosis and liver disease (viral hepatitis, alcoholic consumption >20 g/day for men and >10 g/day for woman, Wilson’s disease, autoimmune, alpha-1-antitrypsin deficiency and drug-induced liver disease). The diagnosis of NAFLD was based on the presence of steatosis by ultrasonography, plus at least one criterion of metabolic syndrome or history of type 2 diabetes.

The diagnosis of HCC was based on the EASL-EORTC Clinical Practice Guidelines [32]. Matched control subjects, aged between 18–40 years, did not shown any evidence of fatty liver at ultrasound. Clinical and anthropometric data were collected at the time of enrolment (Table 1).

### Immunohistochemistry

Formalin-fixed, paraffin embedded sections (4 μm thick) were mounted. For antigen retrieval to detect c-MYC protein, deparaffinized and rehydrated sections were boiled in TRIS-EDTA buffer solution (pH 9) for 20 minutes. For CTR1 protein, deparaffinized and rehydrated sections were microwave-treated in 0.01 M citric acid buffer (pH 6.0), 2 cycles for 5 minutes each at 750 W. The slides were cooled and endogenous peroxidase were blocked with peroxidase block buffer (citric acid 0.04 M, Na2HPO4×2H2O 0.12 M, NaN3 0.03 M and H2O2 at 1.5% v/v) for 10 minutes at room temperature. Then, the sections were incubated for 1 hrs at room temperature with mouse monoclonal antibody anti-c-MYC (clone 9E10; 1:100 diluition, Santa Cruz, Milan) or with rabbit policlonal antibody anti-CTR1 (1:100 diluition, MyBioSource, Milan).

The primary antibodies were visualized using the avidin-biotin-peroxidase complex method (UltraTek HRP Anti-polyvalent, ScyTek, Logan, UT) according to the instruction manual. 3,3′diaminobenzidine was used as the enzyme substrate to observe the specific antibody localization, and Mayer hematoxylin was used as a nuclear counterstain. The score for IHC intensity was scaled as 0 for no IHC signal, 1 for weak, 2 for moderate, and 3 for strong IHC signals. The final score used in the analysis was calculated as already reported [31].

### Cell cultures and treatments

HepaRG cells, a new human hepatoma cell line derived from a human hepatocellular carcinoma, were purchased from Thermo Fisher Scientific (HPRGC10, USA) that provided certificated authentication. These cells hold most hepatic functions, including a considerable expression of uptake transporter proteins in contrast to other hepatic immortalized cell lines [33]. HepaRG cells were maintained in William’s E medium with GlutaMAX supplemented with 10% fetal bovine serum (FBS), 100 U/ml penicillin, 100 μg/ml streptomycin, 5 μg/ml insulin (Sigma-Aldrich) and 5 × 10^−5^ M hydrocortisone hemisuccinate (Sigma-Aldrich). HepG2 were purchased from ATCC (HB-8065). These cells derive from the liver tissue of a 15 year old Caucasian American male with a well differentiated hepatocellular carcinoma, and were grown in Dulbecco’s Modified Eagle’s Medium (DMEM) supplemented with 10% FBS, 1% L-glutamine, 100 U/ml penicillin and 100 μg/ml streptomycin.

All the reagents were purchased from Thermo Fisher Scientific (USA), except as otherwise specified. All cell lines were cultured at 37°C in 5% CO_2_ in a 95% humidified atmosphere and tested for the presence of Mycoplasma. CuSO_4_ (Sigma) has been dissolved in the respective cell culture medium. To induce Omomyc expression 0.25 μg/ml of doxycycline (Doxi) (SIGMA) were used.

### Evaluation of copper content

Cells in hypotonic PBS were lysate by sonication (for 20 s) and then diluted 1:2 (v:v) with 65% nitric acid (ApplicChemPanreac), while serum samples of HD and patients were directly diluted with nitric acid.

After one week of normocaloric diet, all serum samples were collected early in the morning (between 7am and 8am) in fasted subjects.

After copper dosage, sera were frozen immediately at −25°C for few days.

Copper content was assayed by atomic absorption spectroscopy using an AAnalyst 300 instrument equipped with a graphite furnace with platform (HGA800) and an AS-72 autosampler (Perkin-Elmer, Waltham, MA, USA). The results of intracellular copper content obtained (ng Cu) were normalized respect to protein concentration (ng of protein).

### Cell viability assay

Control and CuSO_4_ treated cells were plated in a 96-well microplate and allowed to grow. At the end of copper treatment, the supernatant was aspirated and 100 μL medium mixed with MTS reagent (Promega, WI, USA) were added to each well, according to the manufacturer’s protocol. Wells only containing 200 μl medium were used as blanks and were subtracted as background from each sample. Results obtained in treated cells were expressed as relative respect to untreated cells.

### Viral vectors and infections of Omomyc

The lentiviral plasmid pSLIK-FO harboring a Doxi inducible FlagOmomyc construct was already described [34, 35]. Briefly, Omomyc is an inducible negative dominant interfering MYC bHLHZip dimerization domain. Omomyc has four designed amino acid substitutions that facilitate homodimerization with all three oncogenic MYC proteins (c-Myc, N-Myc and L-Myc) blocking Myc-dependent transcriptional activation. Lentivirus vectors were prepared by co-transfecting HEK293T cells with pSLIK-FO and packaging plasmids PLP1, PLP2 and pMD VSV-G, as described [34, 35]. Viral particles were purified by centrifugation and used for infection in the presence of 4 μg/ml polybrene. Cells infected with Omomyc (HepaRG Omomyc and HepG2 Omomyc) were selected with 50–200 μg/ml hygromycin B (Sigma).

### CTR1 silencing

HepaRG and HepG2 cells were plated, the day before transfection, at 70% confluence. The day after, the culture medium was re-freshed 30 minutes before transfection. 5 pmoles of siCTR1 (Accell SMARTpool, Dharmacon) and scrambled control vector were transfected by using Lipofectamine 2000 reagent (Thermo Fisher Scientific) according to the manufacturer’s instructions. After 72 hrs, MYC, CTR1 and CTR2 mRNA and protein levels were evaluated by Real-Time PCR and Western blotting, respectively.

### RNA extraction and real-time polymerase chain reaction (RT-PCR) analysis

Total RNA extraction was performed using Trizol (Invitrogen) according to the manufacturer’s protocol and 1 μg was reverse transcribed using High-Capacity cDNA Reverse Transcription Kit (Thermo Fisher Scientific, USA) according to the manufacturer’s protocol. Real-Time (RT-PCR) amplification, detection and analysis were performed by 7500 Fast Real-Time PCR System (7500 Software v2.0.5, Applied Biosystems) using Power SYBR™ Green PCR Master Mix (Thermo Fisher Scientific, USA). The results were normalized to the β-actin levels. Relative expression was calculated using the comparative cycle threshold (Ct) method (2−ΔCt, ΔCt = Ct (target gene) - Ct (β-actin)). Data are expressed as fold induction (treated vs untreated) of 2−ΔCt mean ± standard deviation. The following PCR primers were designed by IDT Integrated DNA Technologies and purchased from BIO-FAB research (Rome, Italy): *β-actin*, 5′-GCACTCTTCCAGCCTTCC-3′ and 5′-AGGTCTTTGCGGATGTCCAC-3′; *PCNA*, 5′-TTTCC TGTGCAAAAGACGGA-3′ and 5′-CGTTGAAGAGAGT GGAGTGG-3′; *Cyclin D1*, 5′-ACAAGCTCAAGTGGAA CCTG-3′ and 5′-GAGGGCGGATTGGAAATGAA-3′; *MYC*, 5′-CTCCACACATCAGCACAACT-3′ and 5′-GCC TCTTGACATTCTCCTCG-3′; *CTR1* 5′-CGTAAGTCACA AGTCAGC-3′ and 5′-AGGTACCCGTTGTAGGTC-3′; *CTR2* 5′-CTAGCTTACCCACTTCTC-3′ and 5′-TGG GAATAAGGTGGAGGA-3′, *β-Catenin*, 5′-GCAAGC TCATCATACTGGCT-3′ and 5′-GCATTCCACCAGCT TCTACA-3′; *E-Cadherin* 5′-AGGATCTTGGCTG AGGAT-3′ and 5′-AAGGGGTCTGTCATGGAA-3′.

### Immunoblot analysis

Total protein extraction was performed by homogenizing cells in Ripa lysis buffer containing 1X protease and phosphatase inhibitors cocktail (Thermo Fisher). The homogenates, after 30 min of incubation on ice, were then centrifuged at 13,000 rpm for 30 min at 4°C. The supernatant was then quantified using the Bradford Protein Assay (Bio-Rad, Inc),analysed in denaturing conditions through SDS-PAGE and then transferred and immobilized onto nitrocellulose membranes (Amersham). The membranes were blocked using 5% non-fat dry milk (PanReacApplichem) for 30 min and incubated with the appropriate primary and secondary antibodies. Primary antibodies were the following: β-actin (sc-47778, C4), Cyclin D1 (sc-8396, A-12), MYC (sc-40, 9E10), E-cadherin (sc-8426, G-10), CTR1 (sc-66847, FL-190) all purchased from Santa Cruz Biotechnology; β-catenin (BDBiosciences, 610154, 14/Beta-Catenin), CTR2 (PA5-22961, Thermo Fisher), PCNA (Abcam, Ab 29, PC10) and Flag (Sigma, F3165, M2). Antibody detection was performed by Amersham ECL and Hyperfilm (GE Healthcare Life Sciences). Densitometric analysis of immunoblots was performed by ImageJ64 image processing software for electrophoresis gel analysis.

### Cell cycle analysis

Cells were harvested through trypsinization, washed twice with cold PBS, and subsequently fixed in 70% ethanol at 4°C overnight. After fixation, the cells were washed with PBS and permeabilized with 0.25% Triton X-100 in PBS for 15 min in ice. After washing in cold PBS, cells were incubated in a solution containing 10 mg/mL of RNase and 1 mg/mL of propidium iodide (Sigma-Aldrich, USA) at room temperature for 30 min in the dark. The DNA content was determined using flow cytometry (FACSCanto™ II, BD Biosciences, USA). The percentage of the cells in the G0/G1, S and G2/M phases was determined using FlowJo V10.08 software (Tree Star, Ashland, OR).

### Apoptosis assay by Annexin V-FITC and PI staining

Annexin V Fluorescein (FITC) and Propidium Iodide (PI) double staining assay (Annexin V-FITC Apoptosis detection Kit, eBioscience™) was performed to quantify apoptosis using flow cytometry (FACSCanto™ II, BD Biosciences, USA). Data wereanalyzed using FlowJo V10.08 software (Tree Star Inc., Ashland OR). Analysis allowed discrimination among live (Annexin V-/PI-), necrotic cells (Annexin V-/PI+), late apoptotic (Annexin V+/PI+) and early apoptotic (Annexin V+/PI-).

### Wound-healing migration assay

Cells were plated, in serum free medium, at a density of 100.000 cells on 24-wells plate. The following day, a wound was made with a pipette tip to create a scratch on the monolayer and the serum free medium was changed with medium containing CuSO_4_. Photomicrographs with phase contrast microscope were taken after 48 hrs of treatment.

### Invasion assay

Cells were plated at a density of 40000 cells in the upper compartment of a cell invasion insert (Cultrex Coat BME, Trevigen). The migrated cells into the lower compartment were stained with Crystal Violet (1% Crystal Violet powder in 35% ethanol, w/v) for 10 min and were counted the number of migrated cells in five random fields within the insert. Cells were photographed under an inverted phase-contrast microscope after 48 hrs.

### Chromatin immunoprecipitation (ChIP) assay

Samples for ChIP assays were prepared and analyzed according to MAGnify Chromatin Immunoprecipitation System protocol (Thermo Fischer Scientific, USA). Samples were immunoprecipitated overnight at 4°C with MYC specific antibody 9E10-X, Santa Cruz Biotech, and the promoter region of interest was amplified by RT-PCR. The PCR primers (Thermo Fisher Scientific, USA) designed to amplify the MYC consensus sequence within CTR1 promoter were: Forward 5′-AGCACAT GGCTGTCATTACC-3′ and Reverse 5′-ATGCAACAATC AGTCCAAGC-3′. Relative enrichment was calculated as 2ΔCt(x) - 2ΔCt(b), where ΔCt value was calculated by subtracting the Ct value of the immunoprecipitated sample (x) from the Ct value of the input and the same was calculated for control IgG sample (background (b)).

### Statistical analysis

Results were expressed as mean ± standard deviation (SD). All *in vitro* experiments were performed in triplicate. A 2-tailed paired/unpaired Student *t* test was used to analyze *in vitro* data. The 2-tailed Mann-Whitney test was applied to compare groups of *ex vivo* samples. Statistical significance was assessed by *p*-value (*P*) thresholds: ^*^P < 0.05; ^**^P < 0.01; ^***^P < 0.001. We tested the accuracy of serum Cu concentration for detecting NAFLD-associated HCC by the area under the receiver operating characteristic curve by ROC analysis. The threshold index was identified by ROC analysis, and its sensitivity, specificity and likelihood ratios were calculated. All statistical analyses were performed with Prism software version 6 (GraphPad Software, San Diego, CA).

## SUPPLEMENTARY MATERIALS AND FIGURES


