# Supplementary material for: Copper/MYC/CTR1 interplay: a dangerous relationship in hepatocellular carcinoma
Source: Oncotarget. 2018 Jan 20;9(10):9325–43. doi: 10.18632/oncotarget.24282 (PMC5823635; doi:10.18632/oncotarget.24282)
Supplement: Supplementary file 1 [file oncotarget-09-9325-s001.pdf]

Copper/MYC/CTR1 interplay: a dangerous relationship in hepatocellular carcinoma

SUPPLEMENTARY MATERIALS

Primers sequences

Expression levels of *Cyclin B1* and *Cyclin A* were analysed using SYBR Green- based Real Time RT-PCR. Primers were: *Cyclin B1* 5′-GCAAGCTCATCATACTGG-3′ and 5′-GCATTCCACCAGCTTCTA-3′, *Cyclin A* 5′-GCACCCCTTAAGGATCTTCC-3′ and 5′-TGAACGCAG GCTGTTTACTG-3′.

Antibodies

Cyclin B1 (sc-245, GNS1) and Cyclin A (sc-751, H-432) antibodies were purchased from Santa Cruz Biotechnology.

Detection of intracellular ROS

2′, 7′-dichlorodihydrofluorescein diacetate (H2DCF-DA) fluorescent probe (Thermo Fischer Scientific) was used to identify the intracellular production of reactive oxygen species (ROS). Oxidation of these probes can be detected by monitoring the increase in fluorescence with a flow cytometer using excitation sources and filters appropriate for fluorescein (FITC). HepaRG and HepG2 cells were exposed for 3, 6 and 24 hrs, to 25 and 50 µM H_2O_2 (Sigma-Aldrich), respectively. H_2O_2 concentration was established by performing dose curve analysis with MTS Cell proliferation assay. At the end of copper treatment (20, 35, 50 and 100 µM $CuSO_4$ for 96 hrs) and H_2O_2 exposure, cells were incubated in HBSS and PI (PE) at 37° C per 20 min. Finally, cells were washed with PBS and immediately analyzed by FACS Canto.

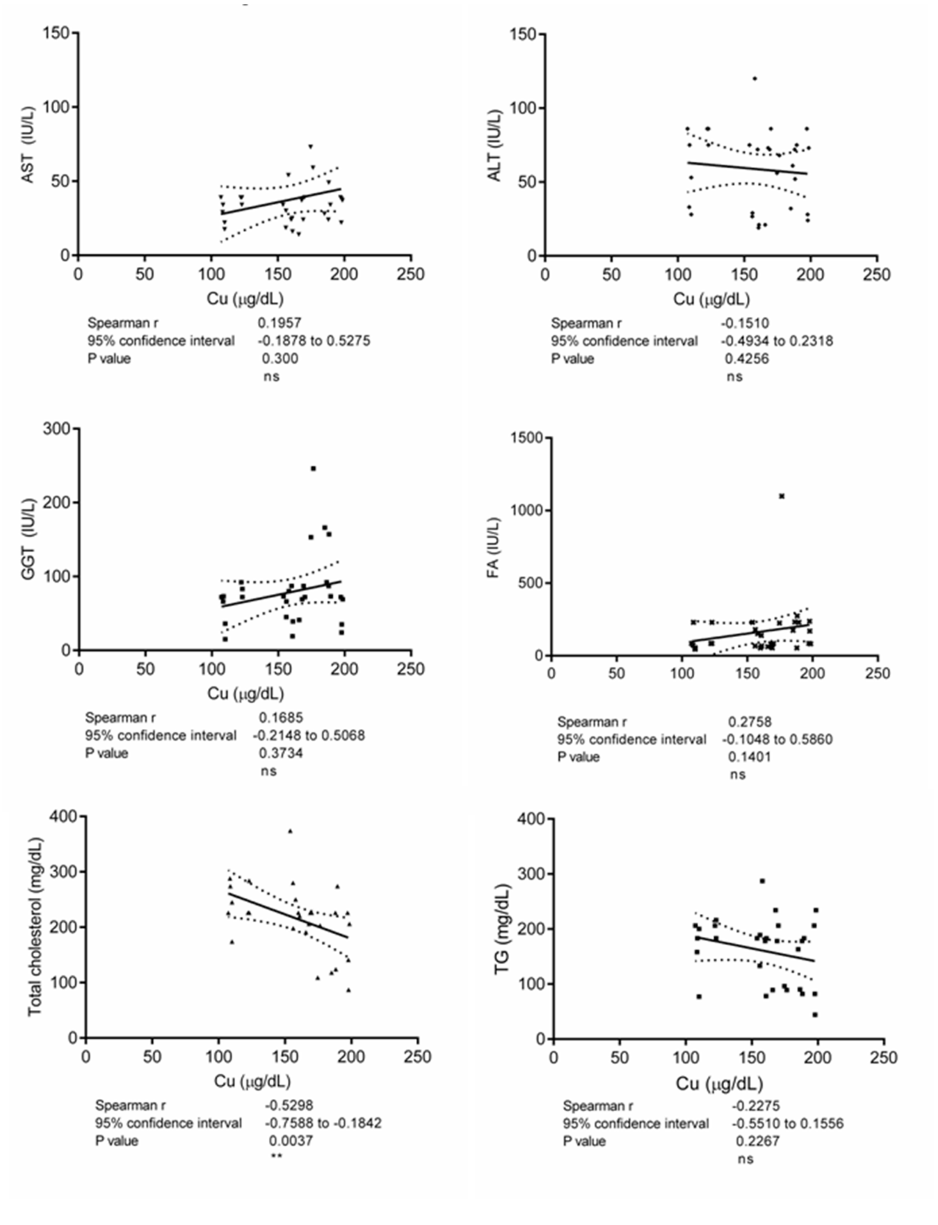

Supplementary Figure 1: Statistical correlations between copper serum levels and biochemical parameters of NAFLD-cirrhotic and -HCC patients: AST, ALT, GGT, FA, total cholesterol and TG. The Spearman's rho, the confidence interval (95%) and P value are reported in each panel.

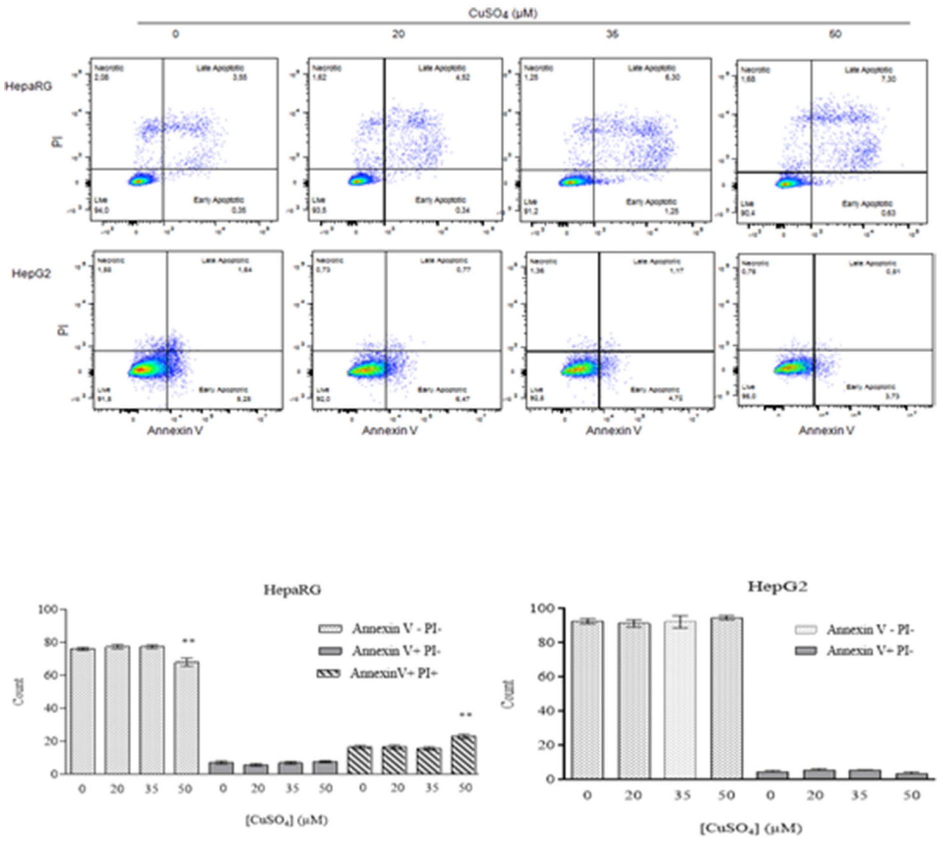

Supplementary Figure 2: Top: Representative images of AnnexinV and PI fluorescent staining of starved cells after 96 hrs of copper treatment. Bottom: Percentage of apoptotic cells in control and copper treated cells evaluated by flow cytometry. Values are represented as mean ± SD. ($^{**}P < 0.01$; $n = 3$).

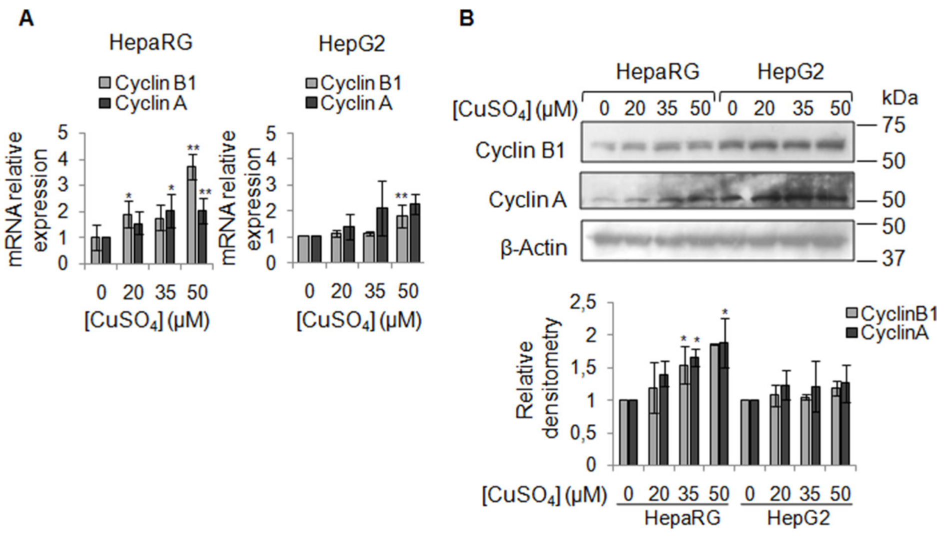

Supplementary Figure 3: Cyclin B1 and Cyclin A expression after copper treatment. (**A**) Relative mRNAs expression of *Cyclin B1* and *Cyclin A* after 96 hrs of copper treatment. (**B**) Representative western blots of Cyclin B1 and Cyclin A (Top) with relative densitometry of three independent experiments (Bottom). All values are expressed as fold mean ± SD. ($^{*}P < 0.05$; $^{**}P < 0.01$).

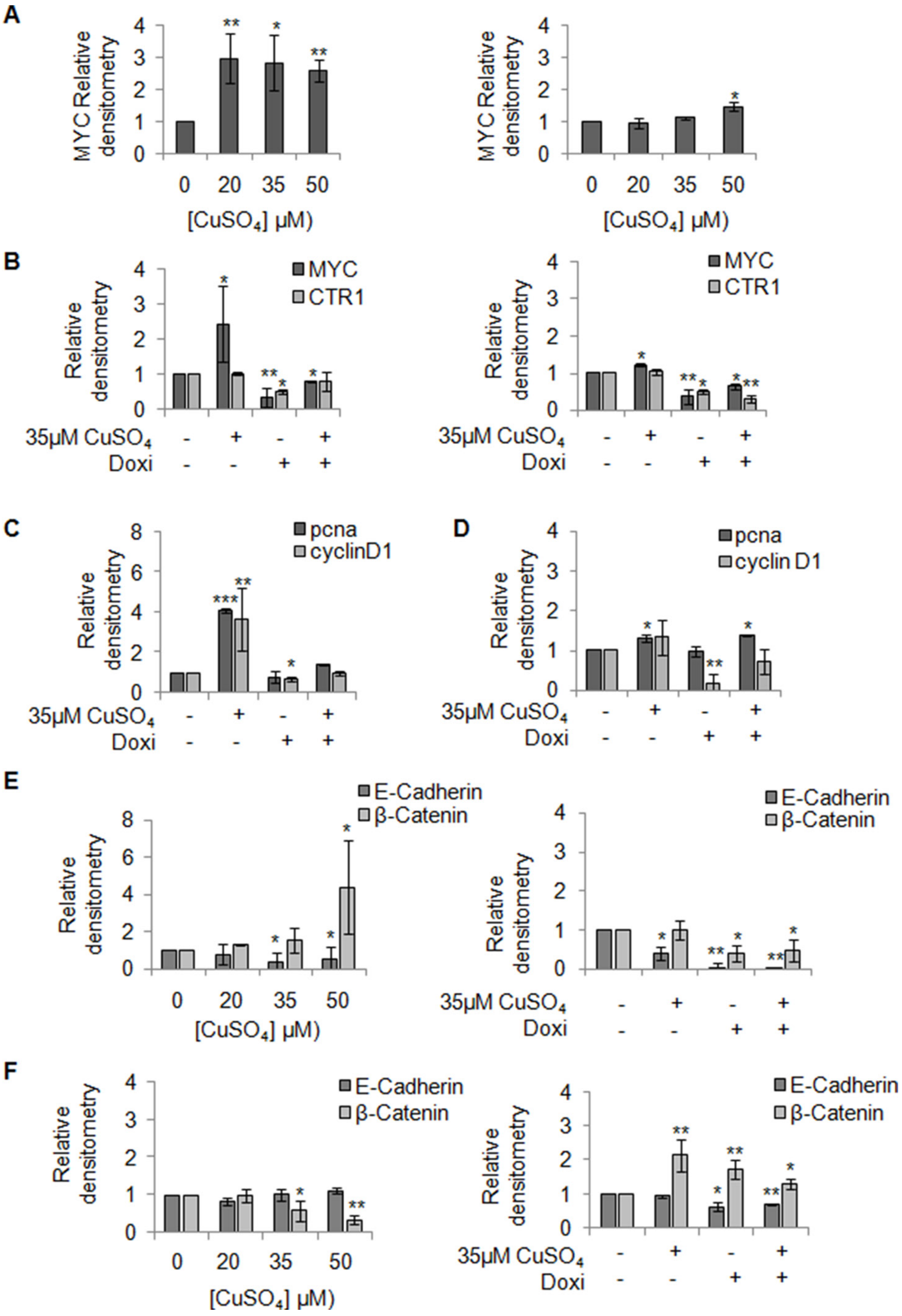

Supplementary Figure 4: Relative densitometry of all the immunoblots reported (**A**) Figure 3B, (**B**) Figure 5A, (**C**) Figure 6B, (**D**) Figure 6D, (**E**, Left) Figure 7C, (E, Right) Figure 7E, (**F**, Left) Figure 8C and (F, Right) Figure 8E. Mean from three independent experiments ± SD. ($^{*}P < 0.05$; $^{**}P < 0.01$ and $^{***}P < 0.001$, respect to control).

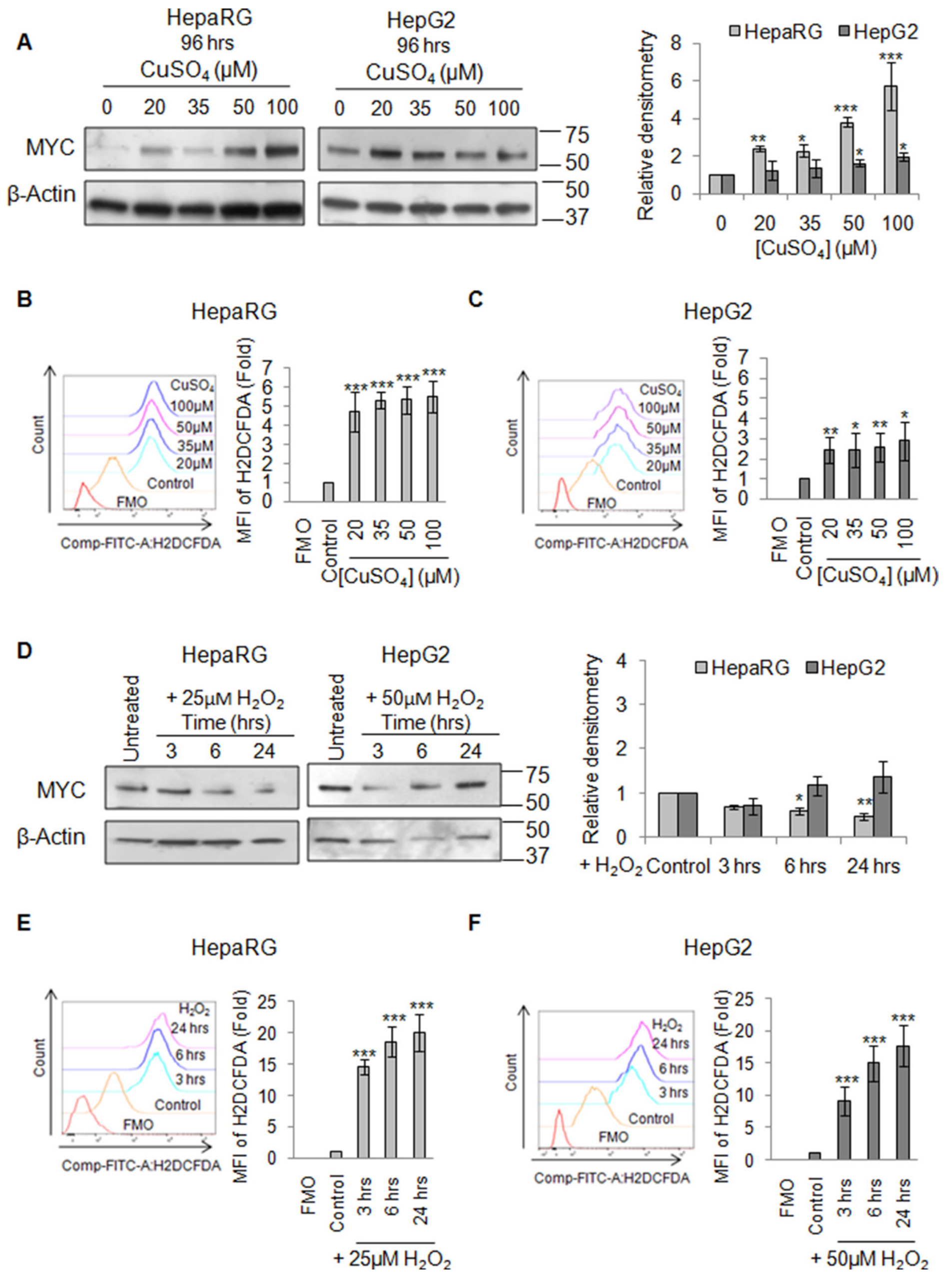

Supplementary Figure 5: (**A**) Representative western blot and relative densitometry of three independent experiments of MYC protein levels in HepaRG (Left) and HepG2 (Right) cells after copper treatment (20, 35, 50 and 100 μM $CuSO_4$ for 96 hrs). (**B** and **C**) Left: Representative histogram overlay showing H2DCFDA (ROS) Mean Fluorescence Intensity (MFI) in HepaRG and HepG2 cells after $CuSO_4$ treatment. Red histogram represents fluorescence-minus-one (FMO). Right: Histograms report relative values of MFI as mean ± SD ($^{*}P < 0.05$, $^{**}P < 0.01$, $^{***}P < 0.001$, respect to untreated cells; $n = 3$). (**D**) Representative western blot of MYC after a time course (3, 6 and 24 hrs) in HepaRG and HepG2 cells, treated with 25 μM and 50 μM of H_2O_2, respectively. (**E** and **F**) Representative histograms overlay showing H2DCFDA (ROS) and relative values of MFI as mean ± SD after H_2O_2 treatment.

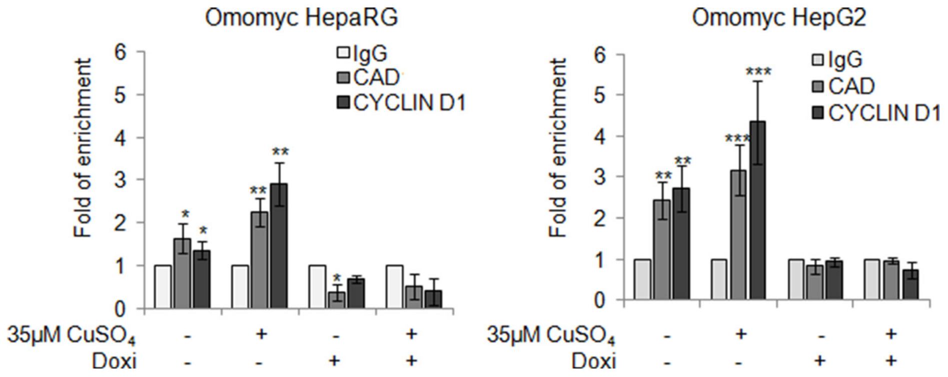

Supplementary Figure 6: MYC binding on target gene promoters. MYC binding to *CAD* (carbamoyl-phosphate synthetase 2) and *Cyclin D1* promoter regions, assayed by ChIP, after induction of Omomyc by doxycyclin (Doxi), and before and after treatment with 35 µM $CuSO_4$. Values are expressed as fold mean ± SD of three independent experiments ($^{*}P < 0.05$ and $^{**}P < 0.01$). IgG was a negative control of ChIP experiments.

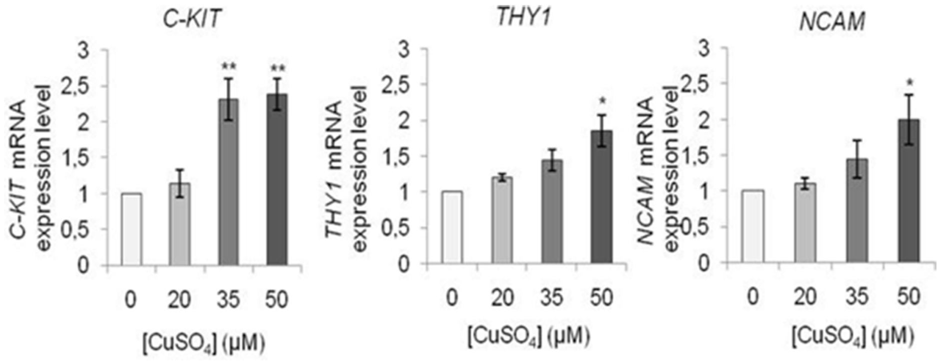

Supplementary Figure 7: Relative mRNA expression levels of *C-KIT*, *THY1* and *NCAM* measured by RT-PCR in HepaRG cells after 96 hrs of $CuSO_4$ treatment. Values are represented as mean ± SD. ($^{*}P < 0.05$ and $^{**}P < 0.01$; $n = 3$).
